# Identification of female-specific genetic variants for metabolic syndrome and its component traits to improve the prediction of metabolic syndrome in females

**DOI:** 10.1186/s12881-019-0830-y

**Published:** 2019-06-06

**Authors:** Sokanha Kong, Yoon Shin Cho

**Affiliations:** Department of Biomedical Science, Hallym University, Chuncheon, Gangwon-do 200-702 Republic of Korea

**Keywords:** Metabolic syndrome (MetS), Single nucleotide polymorphism (SNP), Genome-wide association study (GWAS), Sex specific genetic variants, Genetic risk score (GRS)

## Abstract

**Background:**

Metabolic syndrome (MetS), defined as a cluster of metabolic risk factors including dyslipidemia, insulin-resistance, and elevated blood pressure, has been known as partly heritable. MetS effects the lives of many people worldwide, yet females have been reported to be more vulnerable to this cluster of risks.

**Methods:**

To elucidate genetic variants underlying MetS specifically in females, we performed a genome-wide association study (GWAS) for MetS as well as its component traits in a total of 9932 Korean female subjects (including 2276 MetS cases and 1692 controls). To facilitate the prediction of MetS in females, we calculated a genetic risk score (GRS) combining 14 SNPs detected in our GWA analyses specific for MetS.

**Results:**

GWA analyses identified 14 moderate signals (*P*_*meta*_ < 5X10^− 5^) specific to females for MetS. In addition, two genome-wide significant female-specific associations (*P*_*meta*_ < 5X10^− 8^) were detected for rs455489 in *DSCAM* for fasting plasma glucose (FPG) and for rs7115583 in *SIK3* for high-density lipoprotein cholesterol (HDLC). Logistic regression analyses (adjusted for area and age) between the GRS and MetS in females indicated that the GRS was associated with increased prevalence of MetS in females (*P* = 5.28 × 10^− 14^), but not in males (*P* = 3.27 × 10^− 1^). Furthermore, in the MetS prediction models using GRS, the area under the curve (AUC) of the receiver operating characteristics (ROC) curve was higher in females (AUC = 0.85) than in males (AUC = 0.57).

**Conclusion:**

This study highlights new female-specific genetic variants associated with MetS and its component traits and suggests that the GRS of MetS variants is a likely useful predictor of MetS in females.

**Electronic supplementary material:**

The online version of this article (10.1186/s12881-019-0830-y) contains supplementary material, which is available to authorized users.

## Background

Metabolic syndrome (MetS), defined as a cluster of metabolic risk factors including dyslipidemia, insulin-resistance, and elevated blood pressure, is known to increase the prevalence of other non-communicable diseases such as type 2 diabetes (T2D) and cardiovascular diseases (CVD) [1]. In addition, recent studies reported MetS to be associated with various types of cancer including pancreatic, liver, breast, and colon cancer [2]. The prevalence of MetS is approximately 20 to 30% of the general world population and it is associated with increased morbidity and mortality rates worldwide [2, 3]. Prevalence of MetS is known to increase with age in females; furthermore, females are more prone to MetS due to various cultural factors including stress and low socioeconomic status [3]. The prevalence of cardiovascular diseases that are associated with MetS was shown to be higher than in males, as determined via various meta-analysis studies [4]. A recent systematic review reported that the prevalence of MetS in most countries in Asia-pacific region was higher in females, supported by the data from South Korea in 2007 which described that the prevalence of MetS was higher in females (32.9%) than in males (29%) [5]. Therefore, it is important to thoroughly understand risk factors underlying pathophysiological mechanisms of MetS and the nature of the interaction between gender and the prevalence of this disease.

Until recently, MetS was thought to be a risk factor independent of sex, despite significant research interest in the scientific community; association with various diseases and treatment options have been evaluated irrespective of sex [3]. Since MetS is influenced by not only environmental but also genetic factors, numerous genetic studies have been conducted to gain insight into the genetic basis of MetS and its component traits [6–8]. To the best of our knowledge, however, no female-specific genetic association studies of MetS and its component traits have been conducted in any of ethnic groups including East-Asian as well as European populations. In this study, we sought to identify female-specific genetic variants associated with MetS and its component traits in Korean females by utilizing GWA analysis. In addition, by combining 14 single nucleotide polymorphisms (SNPs) detected from the MetS association analysis, we specifically aimed to investigate whether a genetic risk score (GRS) composed of these SNPs might assist the prediction of MetS in females of South Korean origin.

## Methods

### Subjects

Subjects for the discovery stage were recruited from the KARE (Korea Association Resource) study cohort, a population-based cohort of 8842 participants. The KARE cohort consist of two population-based studies, the Ansung and Ansan cohorts which are located in Gyeonggi Province, close to Seoul, the capital of the Republic of Korea. Details of the participant’s recruitment criteria and the study design are provided elsewhere [9]. A total of 4659 females were present in the KARE study cohort, including 1211 MetS cases and 639 normal controls.

Participants included in the studies used for the replication stage included unrelated Korean participants from the Rural1816, Rural3667, and HEXA (Health Examinee shared control study) cohorts. The Rural1816 cohort includes 1816 subjects, of which 957 are female; of these, 404 MetS cases and 21 normal controls were included in this study. The Rural1816 study combines subjects from the Wonju, Pyeong Chang, Gangneung, Geumsan, and Naju regional cohorts in Korea.

The Rural3667 cohort consists of 3667 subjects of which 2265 females were included in our study; of these, 424 and 330 female subjects were MetS cases and normal controls, respectively. The Rural3667 study combines subjects from the Yangpyeong, Namwon, and Goryeong regional cohorts in Korea. The study design and cohort characteristics of both Rural cohorts have been described previously [9].

A total of 2051 female subjects including 237 MetS cases and 702 normal controls were selected from 3700 subjects of HEXA study that has been described elsewhere [7]. All subjects included for the discovery and replication stages in this study provided written informed consent approved by the local review board. Clinical characteristics of each study cohort are summarized in Table 1.

**Table 1:** Clinical characteristics female subjects in study cohorts. Numbers indicate average and standard deviation for each trait

	KARE	Rural1816	Rural3667	HEXA
^a^N (MetS/contol)	4659 (1211/639)	957 (404/21)	2265 (424/330)	2051 (237/702)
Age (years)	52.6 (±9.0)	60.8 (±6.4)	58.8 (±10.0)	51.6 (±7.7)
Waist circumference (cm)	81.6 (±10.1)	85.1 (±8.5)	82.1 (±8.9)	79.2 (±8.4)
WHR	0.86 (±0.36)	0.89 (±0.06)	0.89 (±0.07)	0.84 (±0.06)
BMI (kg/m^2^)	24.9 (±3.26)	25.2 (±3.4)	24.0 (±3.1)	23.7 (±3.0)
FPG (mg/dL)	82.3 (±25.5)	114.8 (±43.3)	93.0 (±9.3)	91.3 (±26.1)
HDLC (mg/dL)	45.6 (±10.1)	45.4 (±10.6)	46.3 (±10.4)	57.6 (±13.3)
LDLC (mg/dL)	114.4 (±35.4)	133.3 (±35.2)	129.0 (±31.9)	120.4 (±31.5)
TG (mg/dL)	149.0 (±88.9)	169.2 (±110.2)	129.1 (±70.9)	106.6 (±69.4)
TCHL (mg/dL)	191.4 (±35.7)	210.9 (±41.4)	200.6 (±35.7)	198.8 (±35.1)
SBP (mmHg)	124.2 (±20.0)	133.0 (±18.2)	114.7 (±11.7)	119.1 (±14.4)
DBP (mmHg)	81.3 (±11.9)	83.4 (±11.2)	74.4 (±7.5)	75.1 (±9.8)
ALT (IU/L)	23.3 (±17.3)	25.9 (±19.5)	21.1 (±11.4)	20.4 (±12.9)
AST (IU/L)	27.2 (±13.6)	28.2 (±21.2)	25.0 (±8.6)	22.6 (±9.2)
GTP (IU/L)	19.3 (±24.0)	27.7 (±50.6)	17.9 (±16.9)	21.0 (±17.6)
Albumin (g/dL)	4.2 (±0.3)	4.6 (±0.3)	4.4 (±0.2)	4.6 (±0.3)

^a^These numbers indicate the number of subjects used for the association analysis

*WHR* waist-hip ration, *BMI* body mass index, *FPG* fasting plasma glucose, *HDLC* high density lipoprotein cholesterol, *LDLC* low density lipoprotein cholesterol, *TG* triglyceride, *TCHL* total cholesterol, *SBP* systolic blood presure, *DBP* diastolic blood presure, *ALT* aspartate transaminase, *AST* alanine transaminase, *GTP* Glutamyl Transpeptidase

### Genotyping and quality control

For the KARE study subjects, genotyping was carried out using an Affymetrix Genome-Wide Human SNP array 5.0. Details on genotyping quality control (QC) for genotype data have been described previously [9]. For KARE genotype data, SNP imputation was performed to increase the coverage of common variants employing the IMPUTE program (http://mathgen.stats.ox.ac.uk/impute/impute_v2.html) with International HapMap data (phase 2, release 22) of 90 JPT and CHB individuals as the imputation reference panel. Imputed SNPs of poor imputation quality (imputation info score < 0.5, missing gene call rate > 1%, minor allele frequency (MAF) < 0.01, and Hardy-Weinberg equilibrium (HWE) test *P*-value < 1 × 10^− 7^) were excluded [7].

Genotyping of Rural3667 subjects in the replication stage was conducted using Illumina HumanOmni I-Quad vI array. Samples with a genotype missing call rate > 1% and heterozygosity > 30% were excluded from the sample pool. Markers with a missing SNP call rate > 5%, with MAF < 0.01, and with HWE test *P*-value < 1 х 10^− 6^ were eliminated. After this QC process, the remaining 3667 subjects and 747,297 SNPs were included for subsequent analyses.

For the Rural1816 and HEXA study subjects, SNP genotyping was carried out using an Affymetrix Genome-Wide Human SNP array 6.0. Details on genotyping QC for genotype data have been described previously [7].

### Phenotype measurements

In this study, we assessed MetS cases according to International Diabetes Foundation (IDF) criteria. IDF criteria of Korean MetS includes the waist circumference ≥ 85 cm in females (≥ 90 cm in males) plus any two of the following factors: (1) TG ≥ 150 mg/dL, (2) HDLC < 50 mg/dL in females (< 40 mg/dL in males), (3) SBP/DBP ≥ 130/85 mmHg, and (4) FPG ≥ 100 mg/dL [10, 11]. The MetS control group in this study consists of individuals with traits that do not fall into any of the MetS range (i.e., for females, waist circumference < 80 cm, TG < 150 mg/dL, HDLC ≥50 mg/dL, SBP/DBP < 130/85 mmHg, and FPG < 100 mg/dL). Individuals with missing phenotypic values necessary for MetS assessment were not included in the MetS case-control analysis (Table 1).

The phenotypic data of six MetS component traits including waist circumference, fasting plasma glucose (FPG), systolic blood pressure (SBP), diastolic blood pressure (DBP), high density lipoprotein cholesterol (HDLC) and triglycerides (TG) were obtained from the KARE, Rural1816, Rural3667 and HEXA studies (Table 1) [7, 9].

### Statistical analyses

**Fig. 1:**
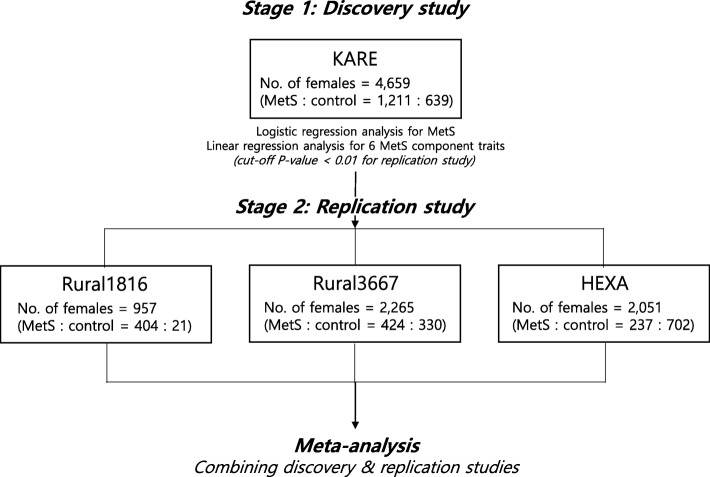
Overall study work flow of two stage genome-wide association analysis for MetS and its component traits

The associations of genetic variants with MetS or six MetS component traits (waist circumference, FPG, SBP, DBP, HDLC, and TG) were carried out after adjustments for age and participants’ areas of recruitment. Logistic (for MetS) or linear (for MetS component traits) regression analyses with the above mentioned adjustments were performed via an additive model using PLINK version 1.07 software (http://zzz.bwh.harvard.edu/plink/) [12]. Because the KARE cohort for the discovery stage as well as both Rural cohorts for the replication stage combines subjects from several regional cohorts, participants’ areas of recruitment were adjusted for the relevant analyses. However, we did not apply principal components for the adjustment of association analyses in this study because previous studies demonstrated that the population stratification is negligible in KARE subjects used in our GWAS discovery stage [7, 9]. Genetic variants with an association *P*-value < 0.01 in the discovery stage were further analyzed in the replication stage, utilizing participants from the Rural1816, Rural3667 and HEXA studies. Meta-analyses were performed to combine discovery stage and replication stage analysis results. This meta-analysis was carried out based on an inverse-variance weighting method using the METAL Program (http://csg.sph.umich.edu/abecasis/metal/index.html) [13] (Fig. 1).

### Generation of the genetic risk score (GRS)

A genetic risk score (GRS) was generated by combining risk alleles of 14 SNPs identified by association analyses for MetS in this study. The risk score of each SNP was attributed based on the risk allele numbers (0, 1, or 2) presented. According to an additive genetic model, each SNP is assumed to be independently associated with risk [14]. Therefore, the GRS of individuals is calculated by summing the risk allele numbers of each of the 14 SNPs. In order to reduce bias and noise, only individuals with no missing genotypes of 14 SNPs were included in analyses employing GRS.

### Construction of a MetS prediction model

The association between the GRS and MetS was tested using logistical regression analysis in females, males, and all subjects combining females and males in the KARE study. To predict the prevalence of MetS in subjects from the KARE study, the logistic regression model was constructed comprising not only GRS, but also two additional variables such as area and age. The constructed model was evaluated by receiver operating characteristics (ROC) analysis and area under the curve (AUC) calculations. Logistical regression analyses were performed using R software, version 3.3.2 (GNU General Public License). The AUC calculation and ROC plot visualization were executed using R’s ModelGood package. A *P*-value of < 0.05 was considered to be significant.

## Results

### Identification of genetic loci for MetS and its component traits

To discover female-specific genetic loci for MetS, we conducted two stage sex-stratified GWAS. In the stage 1, discovery stage, SNPs across the whole genome were tested for their association with MetS in 1211 cases and 639 controls of KARE female subjects. All SNPs showing association *P*-value < 0.01 (15,482 SNPs) from the stage 1 logistic analysis (adjusted for area and age) were taken forward to the stage 2 replication study (Additional file 5: Figure S1). A total of 1065 cases and 1053 controls were included for the replication analysis of female subjects from the Rural1816, Rural3667, and HEXA study cohorts (Fig. 1). Meta-analysis was conducted for SNPs that were validated for their association with MetS (*P*-value < 0.05) in the replication stage by combining association results from discovery and replication stages. Our meta-analysis identified 14 SNPs that fulfill our arbitrary cut-off *P*-value as evidence of moderate association (5 × 10^− 8^ < *P*_meta_ < 5 × 10^− 5^) (Additional file 5: Figure S2). The association of these 14 female MetS signals was not detected in male subjects (Table 2 & Additional file 1: Table S1).

**Table 2:** Metabolic syndrome (MetS) associated loci in females. Discovery stage was GWAS for MetS in each sex-stratified group of KARE study. Overall association results (P_meta_) were obtained from meta-analyses combining Discover (KARE) and Replication (Rural1816, Rural3667 and HEXA) stages

CHR	SNP	BP (GRCh37)	Candidate gene	EA	EAF	Female											Male			Combining female & male		
						Discovery		Replication						Overall								
						KARE		Rural1816		Rural3667		HEXA										
						OR	P_KARE_	OR	P_Rural1816_	OR	P_Rural3667_	OR	P_HEXA_	OR	P_meta_	N	OR	P_meta_	N	OR	P_meta_	N
1	rs2209363	187,163,851	*LINC01036*	C	0.18	0.67	5.32E-04	–	–	0.72	1.27E-02	–	–	0.69	2.11E-05	2496	1.08	3.64E-01	1870	0.88	4.24E-02	4366
2	rs768072	160,233,383	*BAZ2B*	T	0.25	0.72	7.66E-04	–	–	0.72	1.08E-02	–	–	0.72	2.41E-05	2596	0.96	5.72E-01	1922	0.82	2.34E-04	4518
2	rs284544	217,309,111	*SMARCAL1*	A	0.21	1.51	1.65E-04	–	–	1.36	1.77E-02	–	–	1.44	1.06E-05	2572	0.97	7.25E-01	1907	1.15	1.01E-02	4479
2	rs284541	217,368,839	*RPL37A*	T	0.20	1.51	2.56E-04	–	–	1.34	2.90E-02	–	–	1.43	2.66E-05	2531	0.92	3.28E-01	1879	1.12	4.59E-02	4410
2	rs2012243	217,412,087	*LOC101928180*	A	0.21	1.50	1.77E-04	–	–	1.30	3.98E-02	–	–	1.41	2.77E-05	2599	0.93	3.59E-01	1924	1.11	6.45E-02	4523
6	rs10947646	36,881,535	*C6orf89*	G	0.02	0.33	2.66E-04	–	–	–	–	0.35	4.97E-02	0.34	3.46E-05	3196	1.48	9.18E-02	2353	0.78	1.28E-01	5549
8	rs2283113	17,880,243	*PCM1*	A	0.47	1.32	1.42E-03	–	–	1.31	1.15E-02	–	–	1.31	4.77E-05	2524	1.07	3.10E-01	1869	1.18	3.61E-04	4393
9	rs16923249	5,592,145	*PDCD1LG2-RIC1*	A	0.03	0.44	4.26E-04	–	–	–	–	0.39	2.89E-02	0.43	3.46E-05	3214	1.02	9.29E-01	2360	0.73	1.28E-02	5574
13	rs9568558	51,810,953	*FAM124A*	G	0.26	1.33	4.77E-03	–	–	1.39	1.11E-02	1.24	1.15E-01	1.32	4.98E-05	3436	0.99	8.60E-01	2466	1.13	9.69E-03	5902
13	rs9516416	95,103,694	*DCT*	C	0.13	0.68	2.96E-03	0.68	3.71E-01	–	–	0.55	2.16E-03	0.64	2.04E-05	3166	1.00	9.64E-01	2325	0.82	4.17E-03	5491
13	rs6492111	109,055,846	*TNFSF13B-MYO16*	C	0.02	0.35	3.83E-03	0.96	9.66E-01	–	–	0.31	1.74E-03	0.35	3.47E-05	3176	0.75	2.91E-01	2325	0.54	7.51E-04	5501
16	rs4072617	20,178,590	*GPR139*	A	0.19	1.36	3.83E-03	1.41	4.70E-01	1.36	3.02E-02	1.54	2.91E-03	1.41	2.84E-06	3954	0.97	6.36E-01	2857	1.15	4.09E-03	6811
19	rs8107274	37,285,393	*ZNF790*	C	0.03	4.56	5.27E-04	–	–	2.26	1.34E-02	–	–	2.91	4.92E-05	2579	0.85	4.67E-01	1908	1.40	2.94E-02	4487
21	rs2827976	24,600,783	*LOC105372747*	G	0.19	0.69	5.47E-04	0.61	1.50E-01	–	–	0.72	3.74E-02	0.70	1.99E-05	3205	0.97	6.87E-01	2357	0.83	8.28E-04	5562

Information for the SNP ID and chromosomal position is based on NCBI genome build 37/hg19

The ‘-’ sign indicates data not available

*CHR* chromosome, *BP* Physical position (base-pair), *EA* effect allele, *EAF* effect allele frequency, *OR* Odds Ratio, N sample size of meta-analysis combining cases and controls

**Fig. 2:**
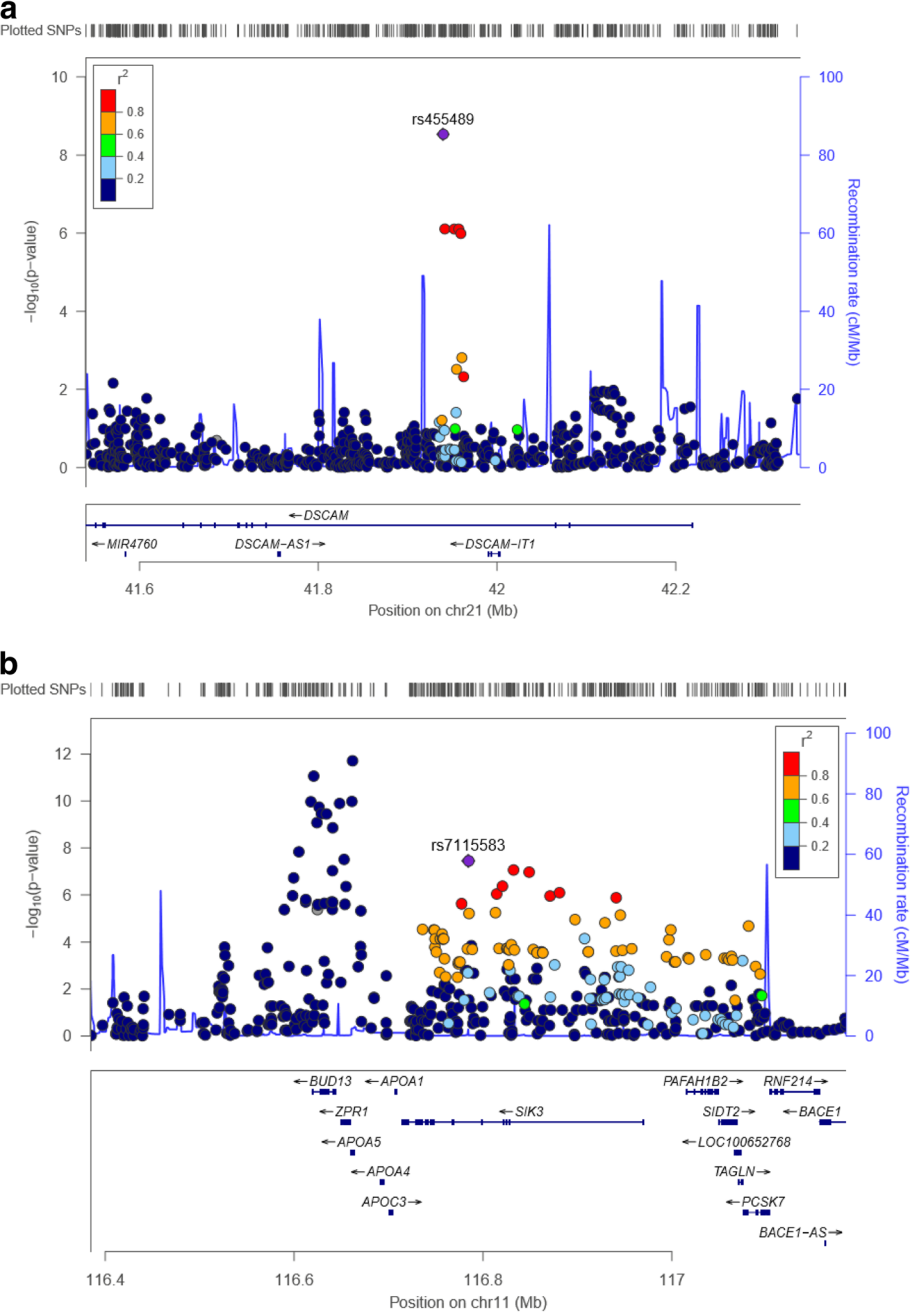
Regional association plots of newly discovered loci for FPG (**a**) and HDLC (**b**) in females. The SNP positions are shown at the top. The trend test -log_10_*P* values are shown for SNPs distributed in a 0.8-Mb genomic region centered on the most strongly associated signal, which is depicted as a purple diamond for the combined results from discovery and replication stages. The locations of known genes in the region are shown at the bottom. The genetic information was obtained from the Human Genome build hg 19; the LD structure was based on the 1000 Genomes ASN data

In order to understand the molecular basis of MetS in females, we also performed sex-stratified GWAS for six MetS component traits including waist circumference, TG, HDLC, SBP, DBP, and FPG. For each trait, genome-wide scan data from a total of 4659 KARE female subjects were tested via linear regression analysis adjusted for area and age in the discovery stage. Next, 5273 female subjects from three studies including Rural1816, Rural 3667, and HEXA were included in the replication stage to validate the signals selected in the discovery stage (*P*_discovery_ < 0.01). SNPs validated in the replication stage (*P*-value < 0.05) were used in the subsequent meta-analysis. Our meta-analyses combining the discovery and replication stages detected two female specific genome-wide significant loci (*P*_meta_ < 5 × 10^− 8^) (Fig. 2) as well as 33 female specific loci showing moderate evidence of association for a given trait (5 × 10^− 8^ < *P*_meta_ < 10^− 5^) (Table 3, Additional file 2: Table S2 & Additional file 5: Figure S3).

**Table 3:** Genetic association results for metabolic syndrome component traits in females. Overall association results were obtained from meta-analyses combining KARE, Rural1816, Rural3667, and HEXA studies

Trait	CHR	SNP	BP (GRCh37)	Candidate gene	EA	EAF	Overall meta (female)					Overall meta (male)			Overall meta (male+female)			Association with MetS
							BETA	SE	*P*_meta_	*P*_Het_	N	BETA	SE	*P*_meta_	BETA	SE	*P*_meta_	*P*_KARE-female_
*Genetic loci showing strong evidence of association for MetS component traits in females (P*_*meta*_ *< 5X10*^*−8*^*)*																		
FPG	21	rs455489	41,939,569	*DSCAM*	C	0.02	6.76	1.14	2.92E-09	0.53	7442	0.51	1.42	7.18E-01	4.03	0.91	9.14E-06	7.07E-02
HDLC	11	rs7115583	116,784,376	*SIK3*	T	0.15	1.32	0.24	3.58E-08	0.20	6905	0.46	0.27	8.79E-02	0.94	0.18	2.30E-07	1.61E-01
*Genetic loci showing moderate evidence of association for MetS component traits in females (5X10*^*− 8*^ *< P*_*meta*_ *< 10*^*−5*^*)*																		
WC	1	rs6683379	223,906,421	*CAPN2*	A	0.48	−0.65	0.14	8.05E-06	0.88	6690	0.09	0.14	5.11E-01	− 0.35	0.11	9.53E-04	3.44E-01
	8	rs921449	34,967,179	*LOC105379369*	A	0.32	0.58	0.13	9.29E-06	0.74	9819	−0.08	0.13	5.31E-01	0.27	0.10	6.65E-03	6.88E-01
	20	rs6029818	40,409,163	*CHD6*	A	0.24	0.63	0.14	8.47E-06	0.85	9779	0.04	0.14	7.58E-01	0.30	0.11	4.43E-03	3.20E-01
FPG	2	rs10171377	77,437,279	*LRRTM4*	G	0.31	−1.13	0.25	8.19E-06	0.54	6556	−0.16	0.32	6.28E-01	− 0.68	0.20	6.98E-04	6.93E-01
	3	rs6801331	124,918,754	*SLC12A8*	T	0.11	1.59	0.36	8.45E-06	0.87	9786	0.30	0.46	5.16E-01	1.04	0.29	2.97E-04	1.10E-02
	8	rs7821492	77,823,083	*ZFHX4/PEX2*	C	0.02	8.02	1.56	2.77E-07	0.13	7202	2.06	1.76	2.42E-01	5.33	1.19	7.33E-06	5.50E-01
	10	rs12783001	65,091,110	*JMJD1C*	C	0.02	6.81	1.50	5.36E-06	0.55	7481	1.37	1.69	4.16E-01	4.32	1.14	1.52E-04	8.35E-01
	11	rs571911	126,481,440	*KIRREL3*	C	0.42	−1.76	0.37	1.94E-06	0.50	7497	−0.28	0.43	5.13E-01	−1.13	0.29	7.87E-05	8.41E-01
	12	rs17125610	62,392,548	*FAM19A2*	A	0.10	2.59	0.58	9.33E-06	0.95	7453	−0.26	0.70	7.11E-01	1.25	0.46	6.23E-03	6.18E-01
SBP	5	rs10474306	89,069,875	*LOC105379076*	G	0.14	1.70	0.38	9.37E-06	0.72	7531	−0.29	0.39	4.60E-01	0.78	0.28	5.19E-03	1.29E-01
	5	rs316224	96,414,272	*LIX1-AS1*	A	0.29	1.30	0.29	9.72E-06	0.76	7660	−0.17	0.30	5.61E-01	0.65	0.21	2.06E-03	3.90E-01
	6	rs393628	5,109,119	*LYRM4*	T	0.09	1.97	0.44	7.75E-06	0.83	6657	0.11	0.48	8.19E-01	1.20	0.33	2.67E-04	4.14E-01
	7	rs4419754	79,337,748	*LOC105375370*	C	0.05	−2.86	0.58	7.30E-07	0.66	6700	−0.53	0.62	3.91E-01	−1.78	0.43	3.20E-05	1.94E-01
	15	rs16973236	82,235,114	*LOC102724001*	G	0.17	1.69	0.38	7.20E-06	0.62	6668	0.23	0.40	5.68E-01	0.99	0.28	3.72E-04	6.21E-02
	17	rs3888658	64,473,272	*PRKCA*	A	0.31	−1.30	0.29	7.17E-06	1.00	7657	0.37	0.30	2.09E-01	−0.51	0.21	1.45E-02	5.46E-01
DBP	12	rs7315532	9,483,063	*LOC105369649*	C	0.37	0.79	0.18	9.89E-06	0.81	7654	−0.11	0.19	5.65E-01	0.40	0.13	2.17E-03	4.30E-01
	14	rs2415841	44,650,411	*LINC02307*	C	0.05	1.82	0.39	2.21E-06	0.82	6647	−0.09	0.43	8.36E-01	1.04	0.29	4.06E-04	4.17E-01
	16	rs1364120	83,740,126	*CDH13*	G	0.32	−0.77	0.17	6.30E-06	0.80	6904	−0.02	0.20	9.10E-01	−0.43	0.13	1.06E-03	2.21E-01
HDLC	11	rs11216315	117,080,640	*PCSK7*	G	0.13	1.17	0.26	6.87E-06	0.78	7649	0.39	0.28	1.64E-01	0.82	0.19	1.84E-05	6.30E-01
	13	rs4883839	69,720,694	na	T	0.28	0.85	0.19	7.87E-06	0.29	7633	0.14	0.20	4.92E-01	0.51	0.14	2.71E-04	1.53E-02
	16	rs6497373	19,460,842	*TMC5*	A	0.16	−1.04	0.23	8.45E-06	0.46	7650	0.02	0.25	9.31E-01	−0.61	0.17	4.15E-04	5.78E-01
	18	rs11082766	47,132,464	*LIPG*	T	0.27	0.96	0.19	6.89E-07	0.27	7649	0.24	0.21	2.45E-01	0.60	0.14	3.39E-05	6.26E-01
	19	rs6508974	41,733,145	*AXL*	C	0.37	0.87	0.18	1.09E-06	0.63	7652	−0.06	0.19	7.39E-01	0.44	0.13	8.21E-04	3.74E-03
	20	rs6046295	19,727,038	*SLC24A3/RIN2*	A	0.05	1.58	0.35	4.40E-06	0.11	9819	−0.34	0.39	3.89E-01	0.66	0.26	1.12E-02	1.13E-01
TG	1	rs3766235	47,049,747	*MKNK1*	C	0.03	17.81	3.95	6.57E-06	0.30	6893	3.46	6.86	6.15E-01	11.45	3.72	2.08E-03	7.11E-01
	1	rs11208004	63,145,439	*DOCK7*	A	0.17	−8.10	1.82	9.13E-06	0.42	6730	−5.56	2.98	6.19E-02	−7.30	1.67	1.27E-05	7.06E-01
	5	rs1092913	10,467,702	*ROPN1L*	G	0.29	7.54	1.46	2.33E-07	0.32	7658	1.69	2.26	4.54E-01	5.08	1.34	1.49E-04	2.09E-01
	6	rs3132722	29,867,174	*LOC105375010*	T	0.26	6.90	1.54	7.61E-06	0.81	6629	4.63	2.51	6.48E-02	6.17	1.42	1.37E-05	2.60E-01
	9	rs1381151	24,957,550	na	A	0.12	9.14	2.05	8.24E-06	0.56	6876	−0.75	3.35	8.24E-01	4.84	1.89	1.02E-02	8.20E-01
	12	rs710698	70,369,918	*MYRFL*	G	0.30	6.85	1.49	4.34E-06	0.52	6664	3.58	2.44	1.42E-01	5.62	1.37	4.27E-05	1.88E-01
	14	rs17124780	52,425,506	*GNG2*	G	0.29	−6.73	1.24	5.52E-08	0.33	9920	−1.09	2.02	5.87E-01	−4.35	1.15	1.55E-04	6.47E-01
	16	rs179604	13,814,473	*SHISA9/ERCC4*	T	0.49	−5.89	1.33	9.47E-06	0.16	7655	0.76	2.05	7.12E-01	−2.73	1.22	2.48E-02	1.58E-01
	22	rs1065314	33,258,288	*SYN3*/*TIMP3*	C	0.06	14.58	2.82	2.41E-07	0.44	6738	0.62	4.55	8.92E-01	8.12	2.57	1.61E-03	3.48E-01

Information for the SNP ID and chromosomal position is based on NCBI genome build 37/hg19

*CHR* chromosome, *BP* Physical position (base-pair), *EA* effect allele, *EAF* effect allele frequency, *SE* standard error, *PHet* heterogeneity p-value, *N* sample size, *WC* waist circumference, *FPG* fasting plasma glucose, *SBP* systolic blood presure, *DBP* diastolic blood presure, *HDLC* high density lipoprotein cholesterol, *TG* triglyceride

Of two loci reaching genome-wide significance, rs455489 located in an intron of the *DSCAM* gene, was significantly associated with FPG levels in females (*P*_*meta-female*_ = 2.92 × 10^− 9^, β = 6.76 ± 1.14), but not in males (*P*_*meta-male*_ = 0.72) (Fig. 2a). The other genome-wide significant locus, rs7115583, located in an intron of the *SIK3* gene, was identified for its association with HDLC levels (*P*_*meta-female*_ = 3.58 × 10^− 8^, β = 1.32 ± 0.24) (Fig. 2b); however, this SNP was shown to have no effect on HDLC levels in males in this study (*P*_*meta-male*_ = 0.09) (Table 3). Of a total of 33 moderate signals, 3 were associated with waist circumference, 6 with FPG, 6 with SBP, 3 with DBP, 6 with HDLC, and 9 with TG (Table 3 & Additional file 2: Table S2). The association of these loci was only detected in females, not in males.

### Generation of a GRS and a MetS prediction model

**Fig. 3:**
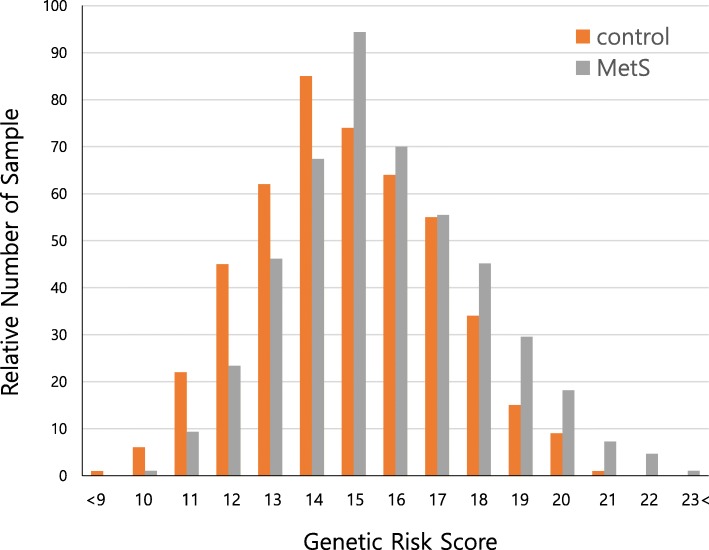
Combined effect of risk alleles on MetS. On the x-axis, each genetic risk score (GRS) category was shown. The y-axis in the histogram indicates the relative number of female subjects in each GRS category. Grey and orange bars represent MetS cases and controls, respectively

Since each genetic variant only confers a modest effect on MetS in females, there is a limitation to MetS prediction in females by applying single variants. To avoid this limitation, we calculated a genetic risk score (GRS) combining risk alleles of 14 SNPs moderately associated with MetS in females. Distribution of the GRS in 912 MetS cases and 473 controls derived from the KARE female subjects indicated that individuals with a high GRS tend to have higher susceptibility to MetS as compared to those with a low GRS (Fig. 3).

In a logistic regression model, GRS was found to be significantly associated with the prevalence of MetS in female subjects from the KARE study (*P* = 4.70 × 10^− 11^). Binary logistic regression adjusted by area and age further increased the association strength between GRS and female MetS (*P* = 5.28 × 10^− 14^). This prediction model for MetS comprising area, age, and GRS as predictor variables implies that a one unit increase in GRS increases the odds of developing MetS in females by a factor of 1.27. On the other hand, GRS was not associated with MetS in male subjects from the KARE study in this model (*P* = 0.33). Although the GRS calculated by this model showed significant association with MetS in all KARE subjects including males and females (*P* = 1.69 × 10^− 5^), the odds of developing MetS was almost negligible (OR = 1.09) (Table 4).

**Table 4:** Logistic regression models for MetS using female-specific GRS

Prediction model for MetS	Study	Sample size (case/control)	Group	Logistic *P*_GRS_-value	OR (95% CI)	AUC
AREA + AGE + GRS	discovery (KARE)	1385 (912/473)	female	5.28E-14	1.27 (1.19–1.35)	0.85
AREA + AGE		1385 (912/473)	female	na	na	0.83
GRS		1385 (912/473)	female	4.70E-11	1.19 (1.13–1.25)	0.60
AREA + AGE + GRS		1076 (503/573)	male	3.27E-01	0.97 (0.92–1.03)	0.57
AREA + AGE + GRS		2461 (1415/1046)	all	1.69E-05	1.09 (1.05–1.13)	0.72

*AUC* area under the curve, *GRS* genetic risk score, *na* not available

**Fig. 4:**
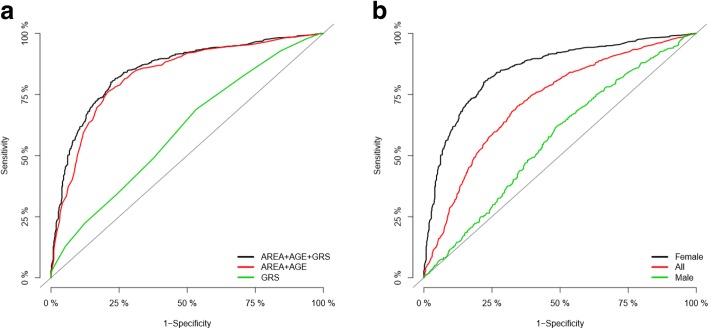
Receiver operating characteristic (ROC) curves for models to predict MetS in the discovery stage (KARE study). (**a**) In KARE females, AUC measured from the ROC curve of a model comprising area, age, and female-specific GRS as predictor variables showed higher value (black) compared to those of other models adjusted with area and age (red) or with only female-GRS (green). (**b**) In a model comprising area, age, and female-GRS, AUC showed higher value in females (black) than in males (green) or all subjects (red)

To evaluate the MetS prediction model, we conducted receiver operating characteristics (ROC) curve analysis employing R’s ModelGood package. In female subjects from the KARE study, the area under the curve (AUC) measured from the ROC curve of a model comprising area, age, and the GRS as predictor variables showed higher values (AUC_area + age + GRS_ = 0.85) compared to those of other models adjusted with area and age (AUC_area + age_ = 0.83) or with only GRS (AUC_GRS_ = 0.60) (Table 4 & Fig. 4a). The model adjusted with area, age, and GRS further effectively predicted MetS in females (AUC_female_ = 0.85) but not in males (AUC_male_ = 0.57) or all subjects (AUC_all_ = 0.72) (Table 4 & Fig. 4b). Taken together, the GRS generated from our association analysis for MetS was a likely predictor for MetS in all female but not in male-containing populations.

## Discussion

Several studies have been previously conducted to identify genetic loci influencing MetS or its component traits [15–19]. However, the total heritability of these traits is not yet fully understood [20]. To explain the missing heritability of these traits, more detailed investigations have been proposed, including analyses of less common variants, sequence-level data, epigenetic data, and environmental exposures [21, 22].

It has been suggested that sex-specific genetic architecture also influences many complex diseases [23, 24]. Thus, genetic studies should consider sex-specific effects in their design and interpretation in order not to fail to detect a significant proportion of the genes that contribute to risk for complex diseases. Although Zabaneh et al. [15] reported a genetic association study for MetS in Indian Asian men, most previous genetic studies for MetS or its component traits were analyzed without consideration of sex-specific effects.

Looking for ways to treat MetS that are more effective for female patients, we aimed to elucidate the female-specific genetic basis for MetS and its component traits in this study. Genome-wide case-control logistic regression analyses in Korean female populations detected 14 genetic variants showing moderate evidence of association for MetS specifically in females, but not in males (Table 2 & Additional file 1: Table S1). To gain statistical power to detect genome-wide significant signals, the recruitment of a larger number of female subjects may be essential. Gene ontology analysis using the DAVID 6.8 functional annotation tool (https://david.ncifcrf.gov/home.jsp) indicated that the function of genes corresponding to the 14 loci associated with MetS were enriched for the positive regulation of T cell proliferation or T cell co-stimulation (Table 5). Further studies will be necessary to gain insight into the functional relevance of these genes to the development of MetS in females.

**Table 5:** Functional annotation results of 16 genes for MetS using DAVID Bioinformatics Resources 6.8

Trait	No of genes	Category	Term	Count	Genes	P-value
MetS	16	GOTERM_BP_DIRECT	GO:0042102~positive regulation of T cell proliferation	2	*TNFSF13B, PDCD1LG2*	0.035
		GOTERM_BP_DIRECT	GO:0031295~T cell costimulation	2	*TNFSF13B, PDCD1LG2*	0.046
		GOTERM_CC_DIRECT	GO:0005829~cytosol	5	*DCT, MYO16, RPL37A, PCM1, RIC1*	0.091

*GO* Gene ontology, *BP* Biological process, *CC* Cellular component

From genome-wide association analysis for 6 MetS component traits in female populations, we were able to detect two signals reaching genome-wide significance. One genetic variant, rs455489 showing association with FPG (*P*_meta-female_ = 2.92 × 10^− 9^, β = 6.76 ± 1.14), is located in an intron of the *DSCAM* (*DS Cell Adhesion Molecule*) gene (Table 3 & Fig. 2a). This result implies that 1 C allele increase in this SNP has an influence on about 6.76 mg/dL rise of FPG in female populations. It has been known that the coding product of the *DSCAM* gene is a member of the immunoglobulin superfamily of cell adhesion molecules (Ig-CAMs) and is involved in human central and peripheral nervous system development [25]. Although this gene is a candidate for Down syndrome and congenital heart disease [26], its involvement in FPG in females needs to be elucidated from further studies.

Another SNP, rs7115583, showed genome-wide significant association with HDLC (*P*_meta-female_ = 3.58 × 10^− 8^, β = 1.32 ± 0.24) in females (Table 3 & Fig. 2b). This result indicates that 1 T allele increase of this SNP results in a 0.24 mg/dL rise in HDLC in females. SNP rs7115583 is located in intron of *SIK3* (*SIK Family Kinase 3*). The stronger associations with HDLC appeared in the *APOA5* (rs2075291) and *BUD13* (rs11216129) genes as compared to the *SIK3* gene. These variants, however, showed strong associations with HDLC in male subjects as well (*P*_meta-male_ = 4.60 × 10^− 13^ for rs2075291; *P*_meta-male_ = 8.42 × 10^− 8^ for rs11216129). Genetic loci in or near *APOA5* and *BUD13* have been associated with HDLC levels in large-scale GWASs [7, 27]. About 164 kb from these loci (*APOA5* and *BUD13*), our results suggest that the *SIK3* locus is the only independent (r^2^ < 0.2) female-specific HDLC signal in this region. It has been reported that the expression of *SIK3* is related to ovarian cancer development [28]. The molecular biological function of this gene has yet to be elucidated.

In addition to the two genome-wide significant signals, 33 loci showed the moderate evidence of association (5 × 10^− 8^ < *P*_meta_ < 10^− 5^) with a given MetS component trait in females. Among those, SNP rs1364120 was found to be moderately associated with DBP and is located in an intron of the *CDH13* (*Cadherin 13*) gene, encoding a member of the cadherin superfamily. This *CDH13* locus has been previously identified as a susceptibility locus influencing blood pressure by genome-wide study in two European populations [29]. It is known that the *CDH13* encoded protein protects vascular endothelial cells from apoptosis due to oxidative stress and is associated with resistance to atherosclerosis [30]. In this study, the minor allele of rs1364120 has a protective effect on DBP (Table 3). The other SNP, rs11082766, showing moderate association with HDLC is located in an intron of the *LIPG* (*Lipase G, Endothelial Type*) gene. The protein encoded by *LIPG* may be involved in lipoprotein metabolism and vascular biology. It is known that this protein has phospholipase and triglyceride lipase activity by hydrolyzing high density lipoproteins (HDL) more efficiently than other lipoproteins [31]. Another SNP, rs3766235 located in an intron of the *MKNK1* (*MAP Kinase Interacting Serine/Threonine Kinase 1*) gene, shows a moderate association with TG. The encoded protein of *MKNK1*, a Ser/Thr protein kinase, is known to be involved in the p38 MAPK signaling pathway as well as regulation of lipid metabolism and insulin signaling cascades, implying its possible role in TG metabolism [32]. The functional relevance of the other genes corresponding to remaining 30 loci moderately associated with traits of interest will be further investigated in future studies.

Since six traits such as waist circumference, FPG, SBP, DBP, HDLC, and TG are used to define MetS; there is speculation that genetic regulation of these traits is closely related to the development of MetS. To gain insight into this hypothesis, we tested the association between a total of 35 loci (2 genome-wide significant signals and 33 moderately associated signals) for MetS component traits and MetS. Except for three SNPs (rs10171377 for FPG, rs4883839 for HDLC, and rs6508974 for HDLC), most signals did not show association with MetS (Table 3). These results indicate that simply combining genetic factors associated with MetS component traits could not predict the development of MetS.

In this study, each allele of risk variants for MetS only confers a modest effect on the risk in females (Table 2). Thus, applying single variants has probably a limited ability for MetS prediction. It has been suggested that a genetic risk score (GRS) combining multiple loci might improve prediction of target disease [33]. In this regard, we specifically aimed to investigate whether the GRS could reliably predict the prevalence of MetS in females. To the best of our knowledge, our study is the first to test the prediction model for MetS using GRS combining single variants associated with MetS specifically in females of East-Asian populations.

We constructed the GRS based on only subjects from the KARE study since some genotype data from the 14 MetS SNPs detected in this study were not available in other studies (including Rural1667, Rural3667, and HEXA). In order to reduce bias and noise, only female individuals with no missing genotypes of the 14 SNPs were included in the construction of the MetS prediction model employing GRS (912 cases and 473 controls). Our logistic model indicated that a GRS combining the 14 MetS SNPs is strongly associated with MetS prevalence in females; an increase in one unit of the GRS accounts for a 1.19 increase in the prevalence of MetS (*P* = 4.70 × 10^− 11^). When this model was adjusted for area (of subject recruitment) and age, the association strength and effect were further increased (*P* = 5.28 × 10^− 14^, OR = 1.27). The AUC of this model, evaluated using receiver operating characteristic (ROC) curve analysis, was 0.85 (AUC_area + age + GRS_ = 0.85) implying that this model comprising area, age, and GRS as predictor variables might be useful for the prediction of MetS in females (Table 4 & Fig. 4a). On the other hand, GRS was not associated with MetS in males (*P* = 0.33), but substantially associated with MetS in populations containing both male and female subjects (*P* = 1.69 × 10^− 5^) (Table 4 & Fig. 4b).

To evaluate if the sex-specific GRS is stronger and more powerful to predict MetS in a group of same sex, we also performed a new meta-analysis for MetS in male participants and identified 10 SNPs showing suggestive evidence of association with MetS in males (5 × 10^− 8^ < *P*_meta_ < 5 × 10^− 5^). The association of these 10 SNPs was validated in replication analyses (Additional file 3: Table S3). Using risk alleles for MetS detected in males, we calculated male-specific GRS. Our logistic analyses demonstrate that male-GRS was strongly associated with MetS in male participants (*P* = 2 × 10^− 16^, OR = 1.35), but just nominally associated in female participants (*P* = 0.013,, OR = 1.08) (Additional file 4: Table S4). To predict MetS in males and females using male-GRS, we carried out ROC analyses and generated AUCs. Our analyses indicate that ROC-AUCs were 0.66 and 0.54 in male and female participants, respectively (Additional file 4: Table S4 and Additional file 5: Figure S4). Taken together, these results strongly imply that sex-specific GRS is a highly effective indicator to predict the MetS development in a population having the same sex.

## Conclusion

In this study, we were able to detect new female-specific genetic variants influencing MetS and its component traits in Korean populations. Our findings including genome-wide significant signals such as *DSCAM* and *SIK3* loci for FPG and HDLC, respectively, as well as several signals moderately associated with traits of interest provide new insights into the underlying sex stratified causes of this cluster of risks. In addition, our study suggests that the GRS based on 14 SNPs detected in our GWA analyses for MetS is a useful predictor of MetS in female but not in male populations.

## Additional files

Additional file 1: **Table S1.** Metabolic syndrome associated loci in females. Association results of these loci were shown in the discovery stage (KARE), replication stage (Rural1816, Rural3667, and HEXA), and overall meta-analysis, respectively. (XLSX 13 kb)

Additional file 2: **Table S2.** Genetic loci associated with MetS component traits in females. Association results of these loci were shown in the discovery stage (KARE), replication stage (Rural1816, Rural3667, and HEXA), and overall meta-analysis, respectively. (XLSX 18 kb)

Additional file 3: **Table S3.** Metabolic syndrome associated loci in males. Association results of these loci were shown in the discovery stage (KARE), replication stage (Rural1816, Rural3667, and HEXA), and overall meta-analysis, respectively. (XLSX 15 kb)

Additional file 4: **Table S4.** Logistic regression model for MetS using male-specific GRS. (XLSX 10 kb)

Additional file 5: **Figure S1.** The Manhattan plot (A) and the QQ plot (B) of discovery stage GWAS for MetS in females. Both plots were generated using the qqman package in R. **Figure S2.** Regional association plots of newly discovered loci for MetS in females. **Figure S3.** Regional association plots of newly discovered loci for MetS component traits in females. **Figure S4.** Receiver operating characteristic (ROC) curves for models to predict MetS in male (A) and female (B) participants of the discovery stage (KARE study). Each AUC was measured from the ROC curve of a model comprising male-specific GRS in KARE males and females, respectively. (PPTX 4229 kb)

## Data Availability

All data generated or analyzed during this study are included in the article and its supplementary files. The GWAS summary results are available in http://web.hallym.ac.kr/~yooncho/index.html. Other dataset used during the current study are available from the corresponding author on reasonable request.

## Abbreviations

AUC: Area under the curve
DBP: Diastolic blood presure
FPG: Fasting plasma glucose
GRS: Genetic risk score
GWAS: Genome-wide association study
HDLC: High density lipoprotein cholesterol
HEXA: Health Examinee shared control study
KARE: Korea Association Resource study
MetS: Metabolic syndrome
ROC: Receiver operating characteristics
SBP: Systolic blood presure
SNP: Single nucleotide polymorphism
TG: Triglyceride

## References

[1] Cornier MA, Dabelea D, Hernandez TL, Lindstrom RC, Steig AJ, Stob NR, Van Pelt RE, Wang H, Eckel RH (2008). The metabolic syndrome. Endocr Rev.

[2] O'Neill S, O'Driscoll L (2015). Metabolic syndrome: a closer look at the growing epidemic and its associated pathologies. Obesity reviews : an official journal of the International Association for the Study of Obesity.

[3] Pucci G, Alcidi R, Tap L, Battista F, Mattace-Raso F, Schillaci G (2017). Sex- and gender-related prevalence, cardiovascular risk and therapeutic approach in metabolic syndrome: a review of the literature. Pharmacol Res.

[4] Santilli F, D'Ardes D, Guagnano MT, Davi G (2017). Metabolic syndrome: sex-related cardiovascular risk and therapeutic approach. Curr Med Chem.

[5] Ranasinghe P, Mathangasinghe Y, Jayawardena R, Hills AP, Misra A (2017). Prevalence and trends of metabolic syndrome among adults in the asia-pacific region: a systematic review. BMC Public Health.

[6] DeMenna J, Puppala S, Chittoor G, Schneider J, Kim JY, Shaibi GQ, Mandarino LJ, Duggirala R, Coletta DK (2014). Association of common genetic variants with diabetes and metabolic syndrome related traits in the Arizona insulin resistance registry: a focus on Mexican American families in the southwest. Hum Hered.

[7] Kim YJ, Go MJ, Hu C, Hong CB, Kim YK, Lee JY, Hwang JY, Oh JH, Kim DJ, Kim NH (2011). Large-scale genome-wide association studies in east Asians identify new genetic loci influencing metabolic traits. Nat Genet.

[8] Mohlke KL, Boehnke M, Abecasis GR (2008). Metabolic and cardiovascular traits: an abundance of recently identified common genetic variants. Hum Mol Genet.

[9] Cho YS, Go MJ, Kim YJ, Heo JY, Oh JH, Ban HJ, Yoon D, Lee MH, Kim DJ, Park M (2009). A large-scale genome-wide association study of Asian populations uncovers genetic factors influencing eight quantitative traits. Nat Genet.

[10] Huang PL (2009). A comprehensive definition for metabolic syndrome. Dis Model Mech.

[11] Yoon YS, Lee ES, Park C, Lee S, Oh SW (2007). The new definition of metabolic syndrome by the international diabetes federation is less likely to identify metabolically abnormal but non-obese individuals than the definition by the revised national cholesterol education program: the Korea NHANES study. Int J Obes.

[12] Purcell S, Neale B, Todd-Brown K, Thomas L, Ferreira MA, Bender D, Maller J, Sklar P, de Bakker PI, Daly MJ (2007). PLINK: a tool set for whole-genome association and population-based linkage analyses. Am J Hum Genet.

[13] Willer CJ, Li Y, Abecasis GR (2010). METAL: fast and efficient meta-analysis of genomewide association scans. Bioinformatics.

[14] Horne BD, Anderson JL, Carlquist JF, Muhlestein JB, Renlund DG, Bair TL, Pearson RR, Camp NJ (2005). Generating genetic risk scores from intermediate phenotypes for use in association studies of clinically significant endpoints. Ann Hum Genet.

[15] Zabaneh D, Balding DJ (2010). A genome-wide association study of the metabolic syndrome in Indian Asian men. PLoS One.

[16] Kristiansson K, Perola M, Tikkanen E, Kettunen J, Surakka I, Havulinna AS, Stancakova A, Barnes C, Widen E, Kajantie E (2012). Genome-wide screen for metabolic syndrome susceptibility loci reveals strong lipid gene contribution but no evidence for common genetic basis for clustering of metabolic syndrome traits. Circ Cardiovasc Genet.

[17] Kraja AT, Vaidya D, Pankow JS, Goodarzi MO, Assimes TL, Kullo IJ, Sovio U, Mathias RA, Sun YV, Franceschini N (2011). A bivariate genome-wide approach to metabolic syndrome: STAMPEED consortium. Diabetes.

[18] Zhu Y, Zhang D, Zhou D, Li Z, Li Z, Fang L, Yang M, Shan Z, Li H, Chen J (2017). Susceptibility loci for metabolic syndrome and metabolic components identified in Han Chinese: a multi-stage genome-wide association study. J Cell Mol Med.

[19] Lin E, Kuo PH, Liu YL, Yang AC, Tsai SJ (2017). Detection of susceptibility loci on APOA5 and COLEC12 associated with metabolic syndrome using a genome-wide association study in a Taiwanese population. Oncotarget.

[20] Lander ES (2011). Initial impact of the sequencing of the human genome. Nature.

[21] Shin JM, Lim W, Lee KM, Won JI, Jung DW, Nho CW, Kang KD, Yoon JH, Cho YS (2017). Disease-miRNAdb: a manually-curated database for the investigation of the microRNA-human disease relationship. Genes Genomics.

[22] Witte JS (2010). Genome-wide association studies and beyond. Annu Rev Public Health.

[23] Weiss LA, Pan L, Abney M, Ober C (2006). The sex-specific genetic architecture of quantitative traits in humans. Nat Genet.

[24] Ober C, Loisel DA, Gilad Y (2008). Sex-specific genetic architecture of human disease. Nat Rev Genet.

[25] Yamagata M, Sanes JR (2008). Dscam and sidekick proteins direct lamina-specific synaptic connections in vertebrate retina. Nature.

[26] Yamakawa K, Huot YK, Haendelt MA, Hubert R, Chen XN, Lyons GE, Korenberg JR (1998). DSCAM: a novel member of the immunoglobulin superfamily maps in a Down syndrome region and is involved in the development of the nervous system. Hum Mol Genet.

[27] Spracklen CN, Chen P, Kim YJ, Wang X, Cai H, Li S, Long J, Wu Y, Wang YX, Takeuchi F (2017). Association analyses of east Asian individuals and trans-ancestry analyses with European individuals reveal new loci associated with cholesterol and triglyceride levels. Hum Mol Genet.

[28] Charoenfuprasert S, Yang YY, Lee YC, Chao KC, Chu PY, Lai CR, Hsu KF, Chang KC, Chen YC, Chen LT (2011). Identification of salt-inducible kinase 3 as a novel tumor antigen associated with tumorigenesis of ovarian cancer. Oncogene.

[29] Org E, Eyheramendy S, Juhanson P, Gieger C, Lichtner P, Klopp N, Veldre G, Doring A, Viigimaa M, Sober S (2009). Genome-wide scan identifies CDH13 as a novel susceptibility locus contributing to blood pressure determination in two European populations. Hum Mol Genet.

[30] Niermann T, Kern F, Erne P, Resink T (2000). The glycosyl phosphatidylinositol anchor of human T-cadherin binds lipoproteins. Biochem Biophys Res Commun.

[31] Edmondson AC, Brown RJ, Kathiresan S, Cupples LA, Demissie S, Manning AK, Jensen MK, Rimm EB, Wang J, Rodrigues A (2009). Loss-of-function variants in endothelial lipase are a cause of elevated HDL cholesterol in humans. J Clin Invest.

[32] Waskiewicz AJ, Flynn A, Proud CG, Cooper JA (1997). Mitogen-activated protein kinases activate the serine/threonine kinases Mnk1 and Mnk2. EMBO J.

[33] Hung CF, Breen G, Czamara D, Corre T, Wolf C, Kloiber S, Bergmann S, Craddock N, Gill M, Holsboer F (2015). A genetic risk score combining 32 SNPs is associated with body mass index and improves obesity prediction in people with major depressive disorder. BMC Med.

