# Supplementary material for: Identification of female-specific genetic variants for metabolic syndrome and its component traits to improve the prediction of metabolic syndrome in females
Source: BMC Med Genet. 2019 Jun 6;20:99. doi: 10.1186/s12881-019-0830-y (PMC6555714; doi:10.1186/s12881-019-0830-y)
Supplement: Supplementary file 5 — Figure S1. The Manhattan plot (A) and the QQ plot (B) of discovery stage GWAS for MetS in females. Both plots were generated using the qqman package in R. Figure S2. Regional association plots of newly discovered loci for MetS in females. Figure S3. Regional association plots of newly discovered loci for MetS component traits in females. Figure S4. Receiver operating characteristic (ROC) curves for models to predict MetS in male (A) and female (B) participants of the discovery stage (KARE study). Each AUC was measured from the ROC curve of a model comprising male-specific GRS in KARE males and females, respectively. (PPTX 4229 kb) [file 12881_2019_830_MOESM5_ESM.pptx]

## Slide 1
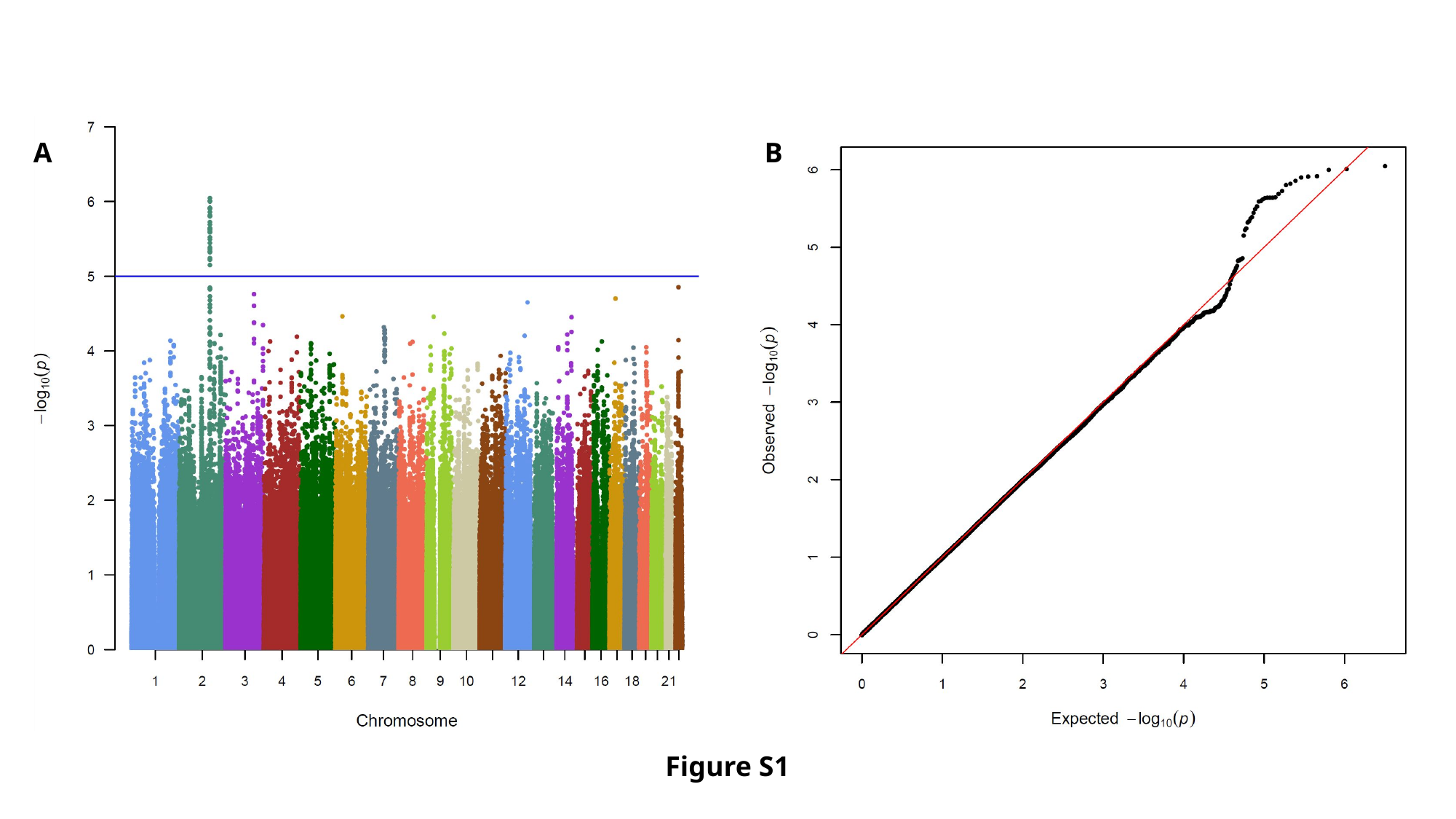

A
B
Figure S1

## Slide 2
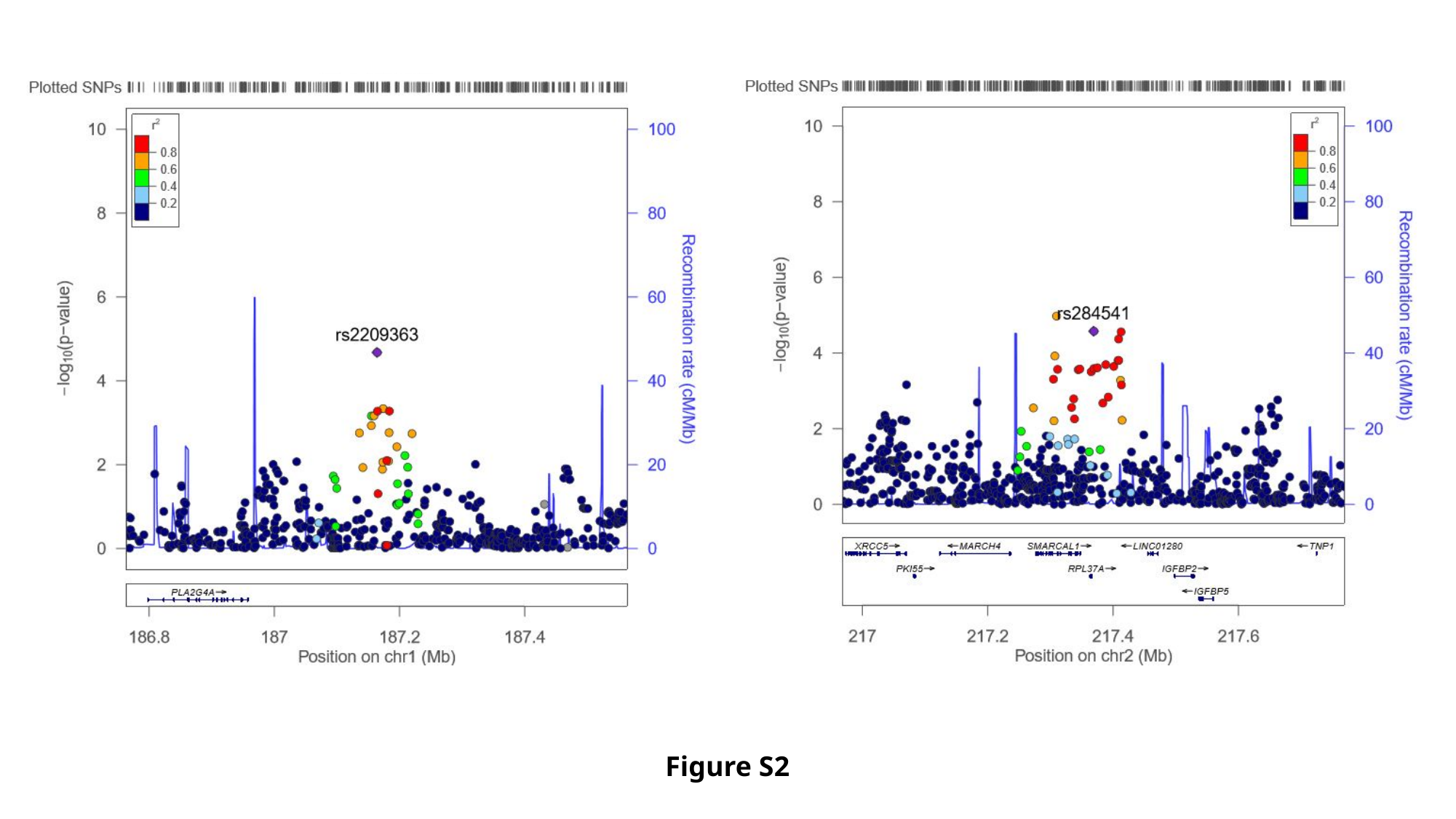

Figure S2

## Slide 3
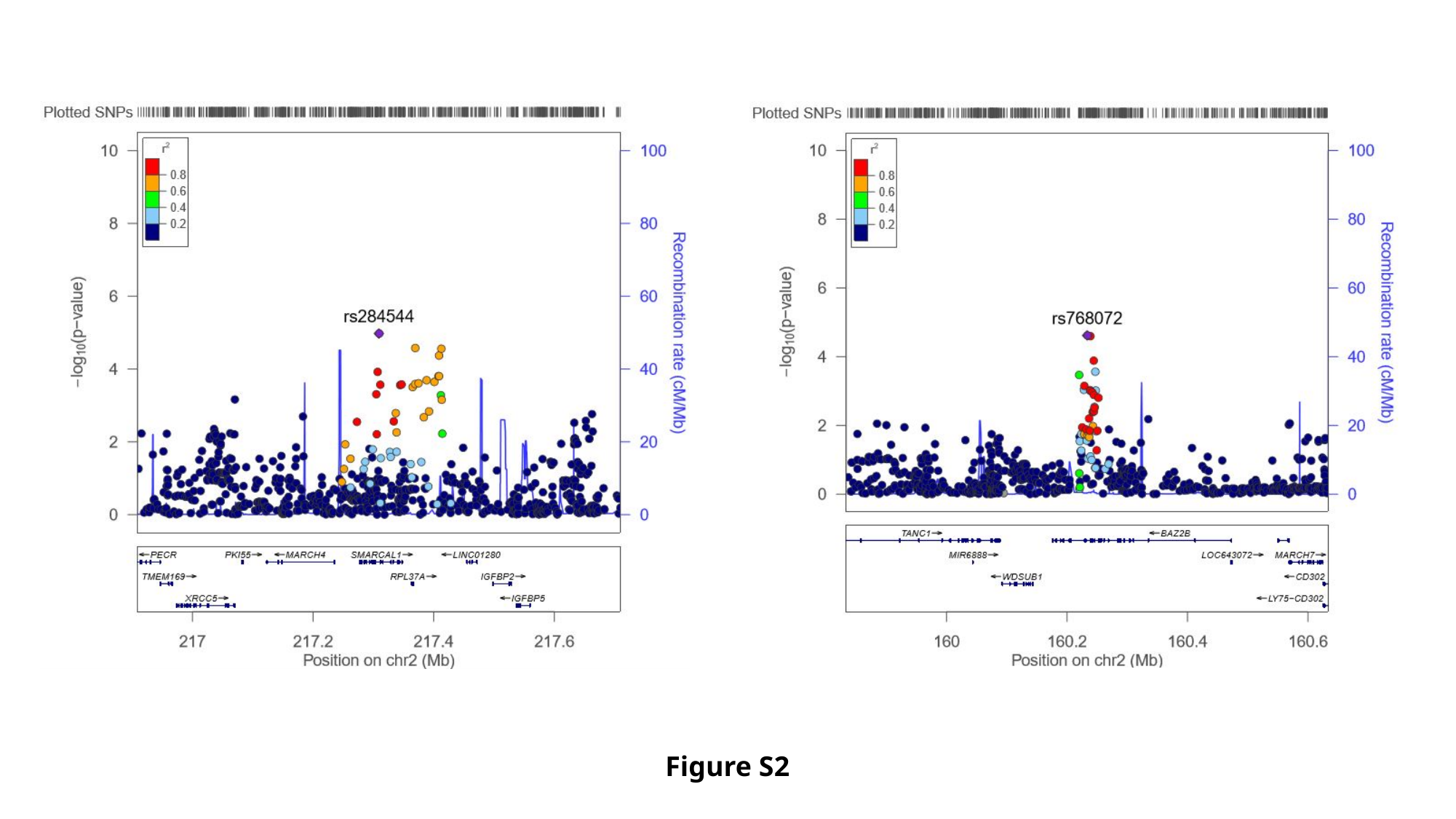

Figure S2

## Slide 4
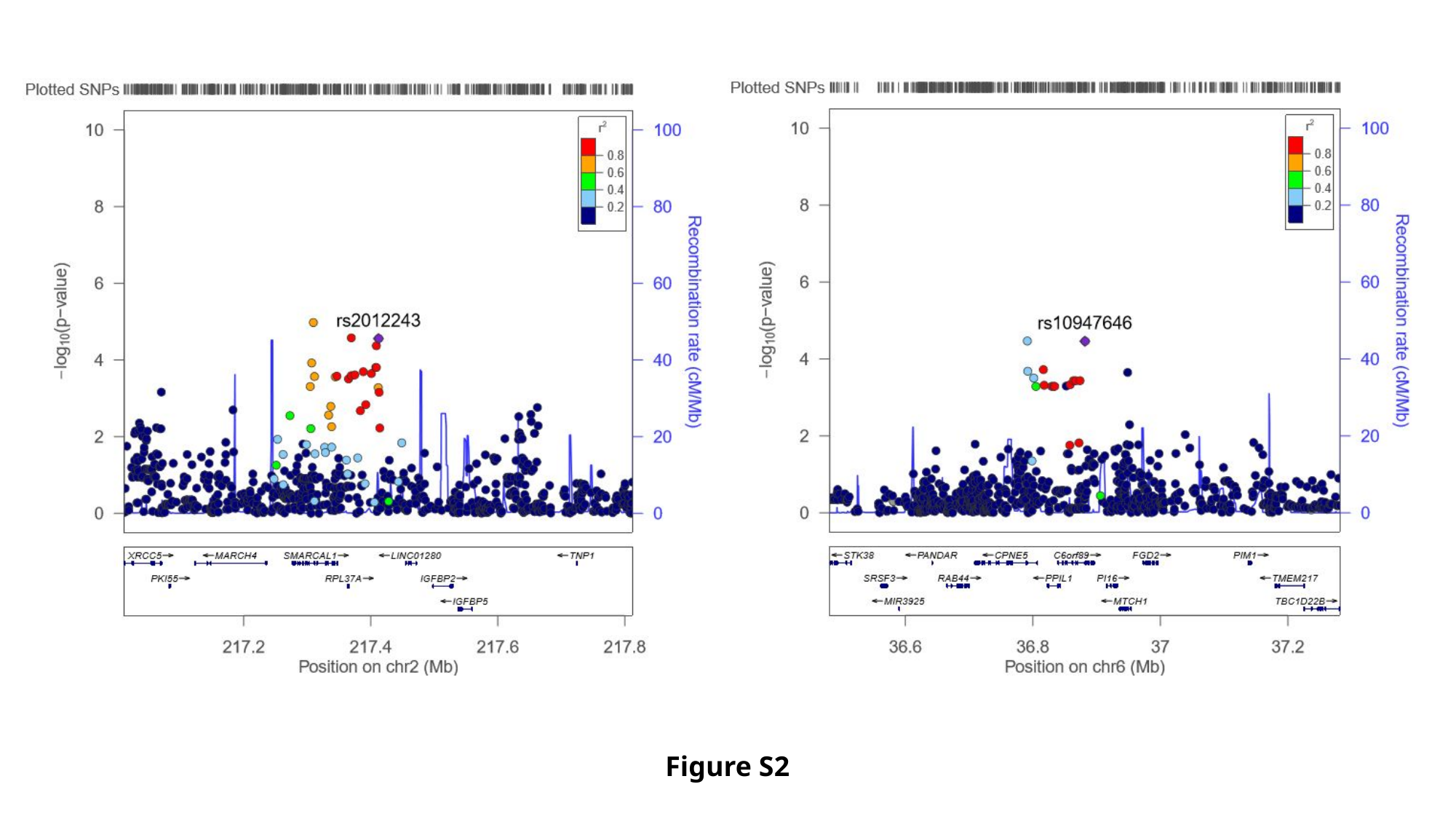

Figure S2

## Slide 5
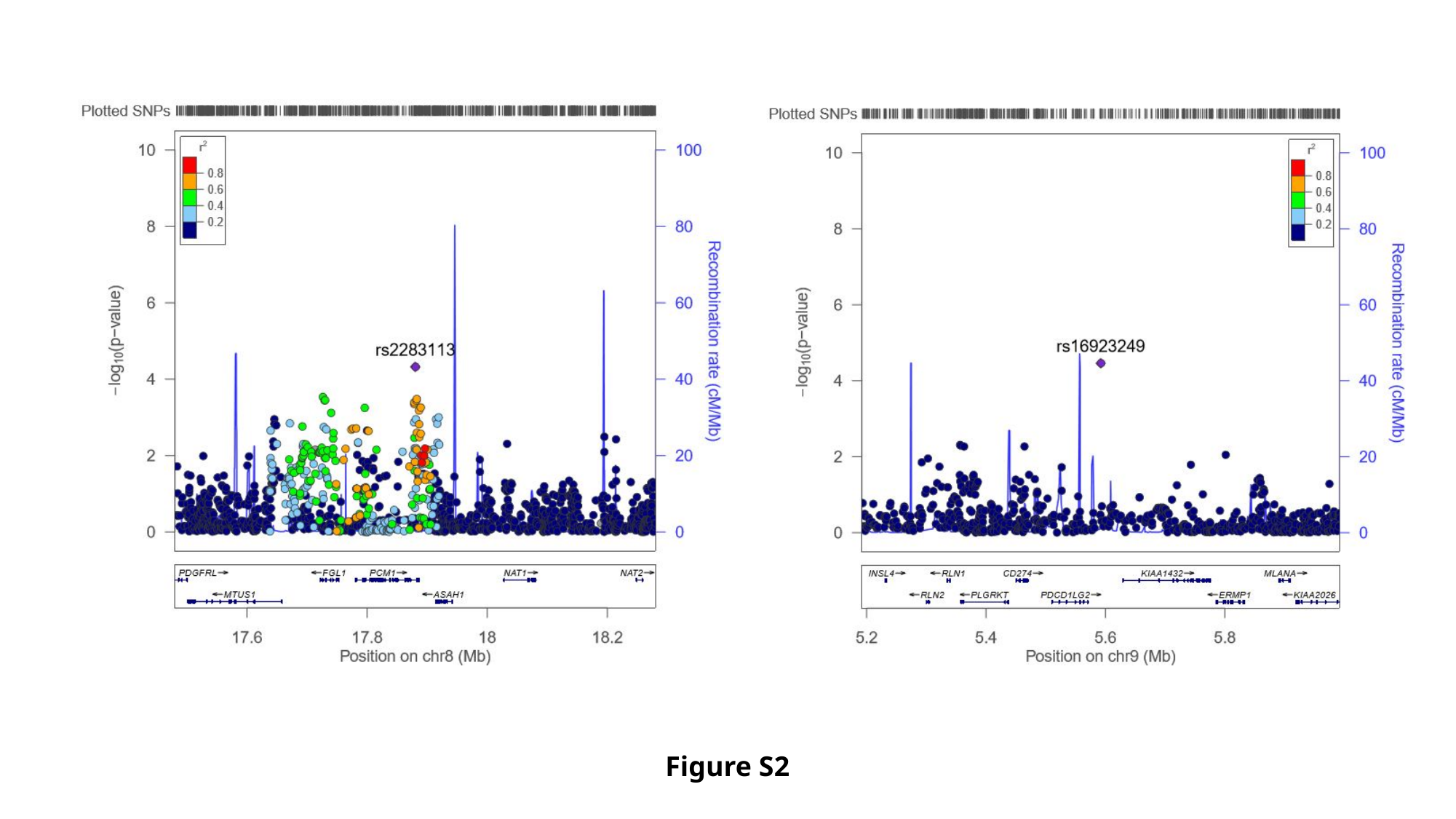

Figure S2

## Slide 6
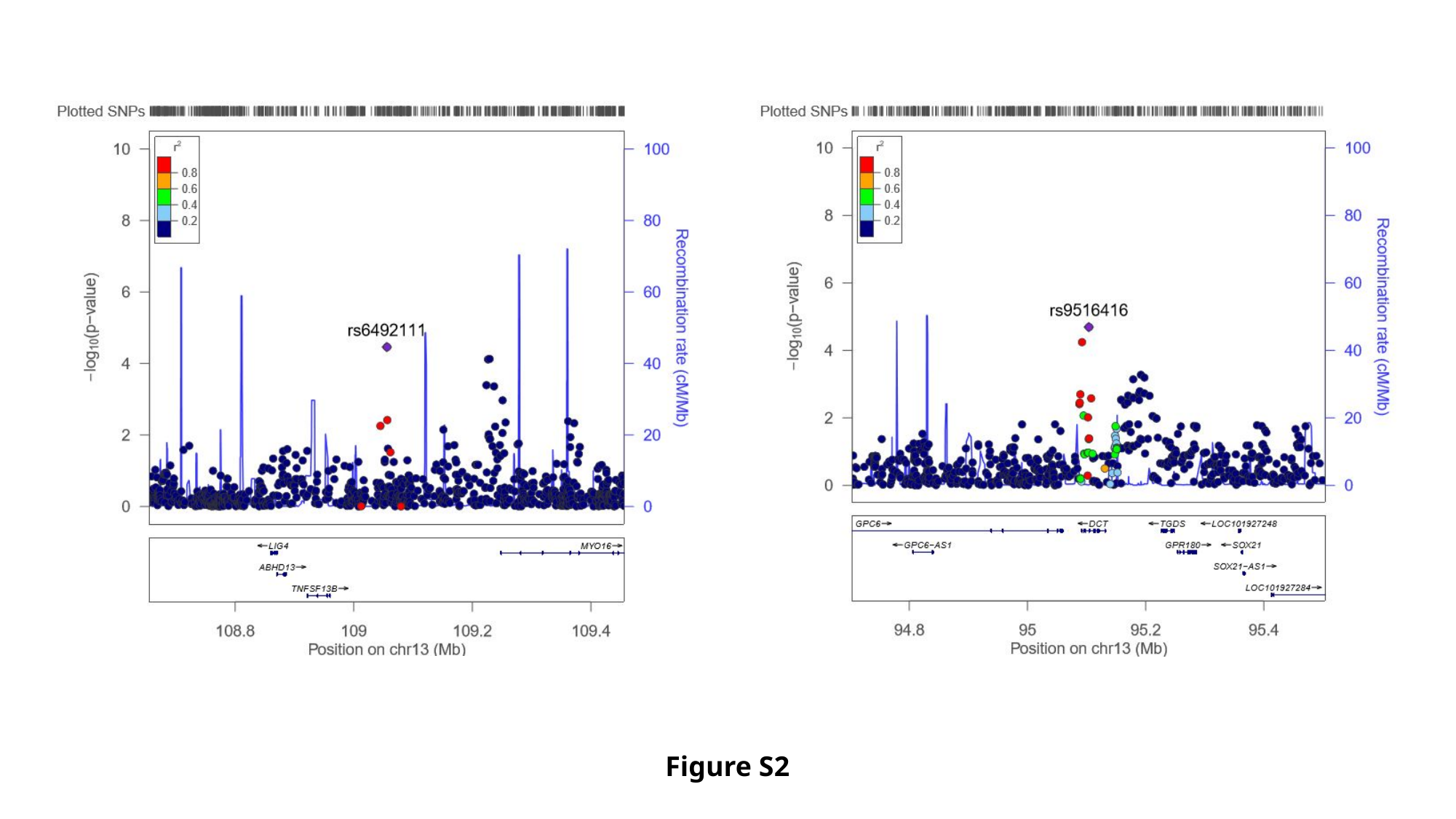

Figure S2

## Slide 7
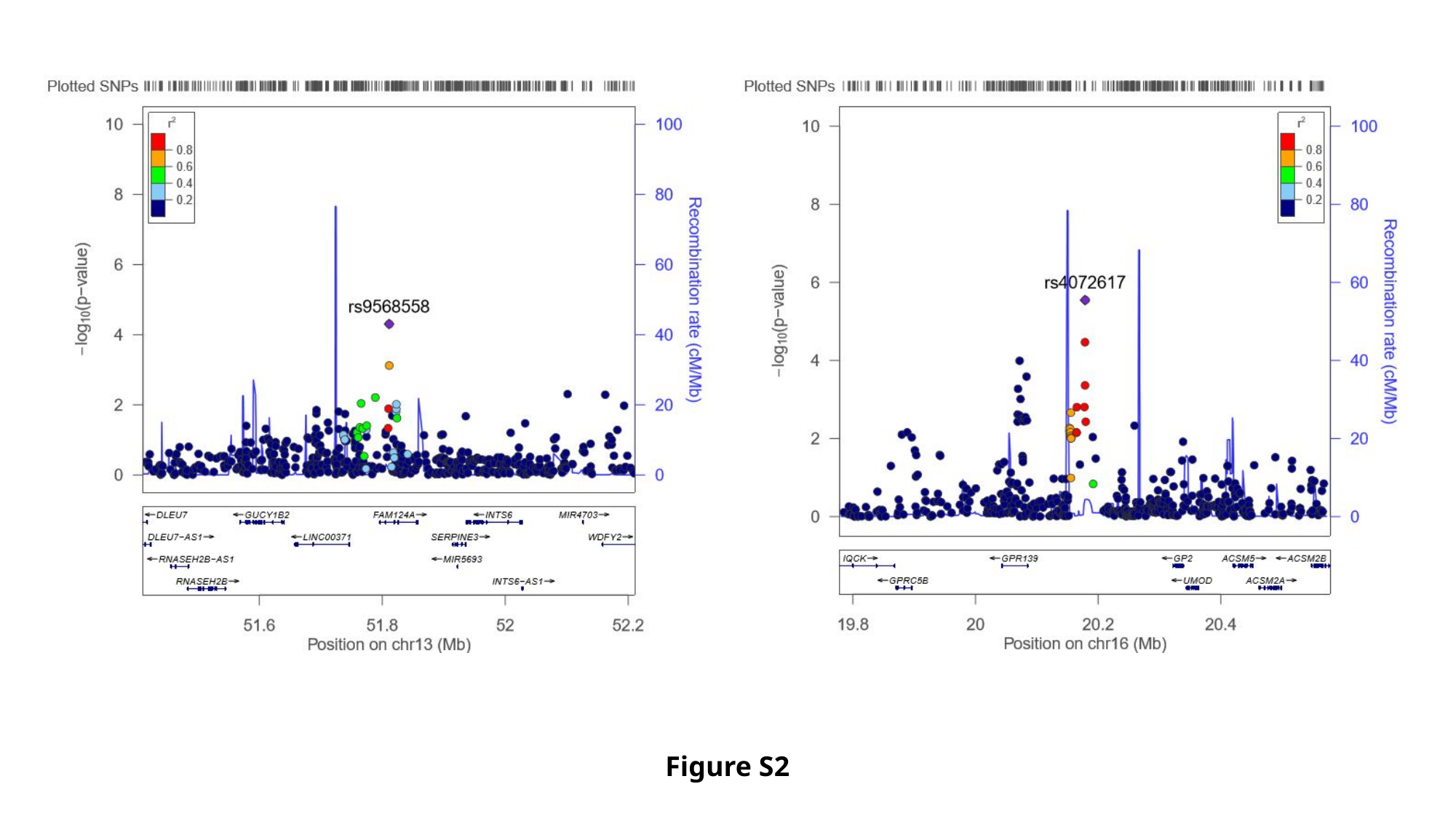

Figure S2

## Slide 8
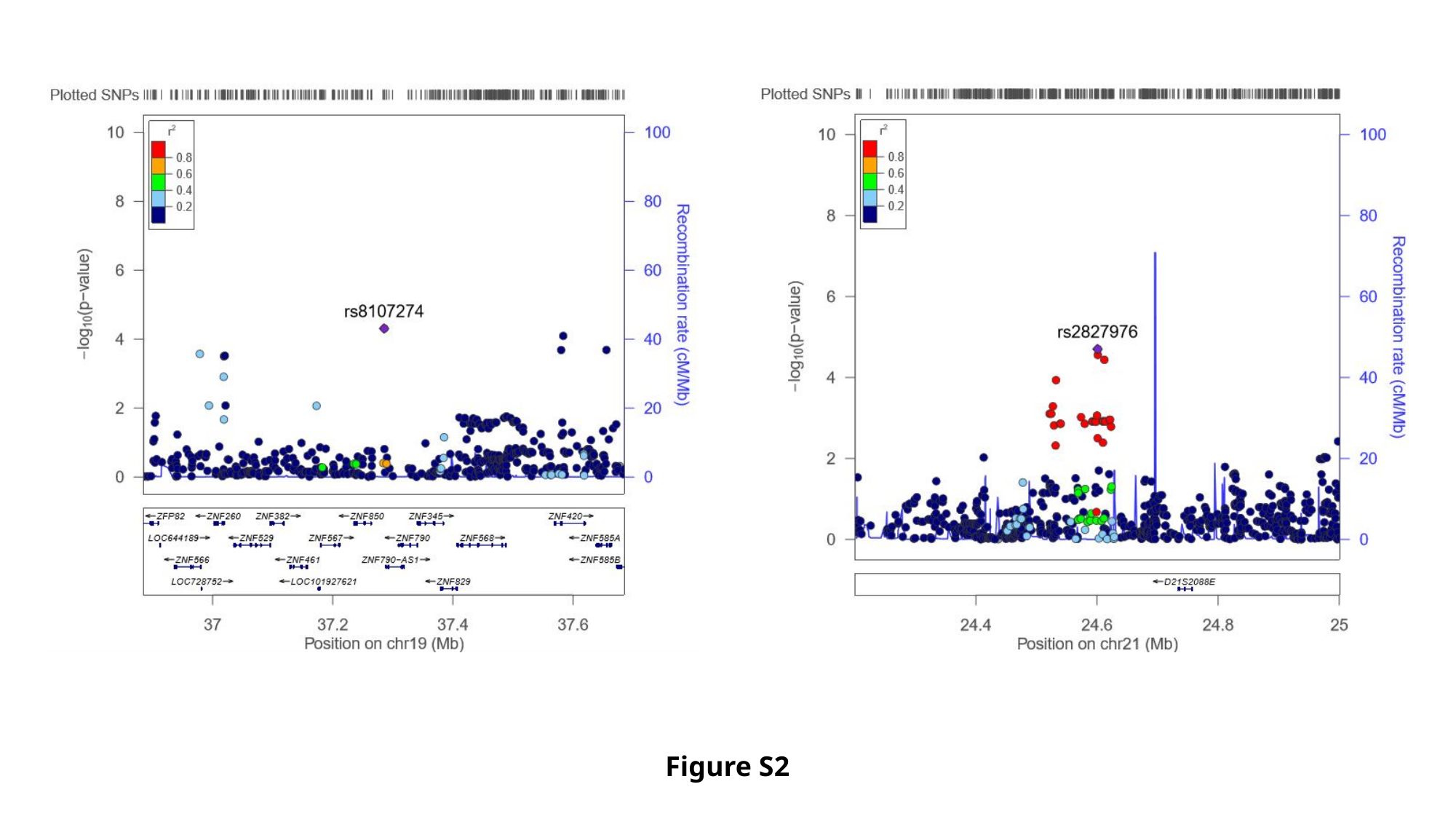

Figure S2

## Slide 9
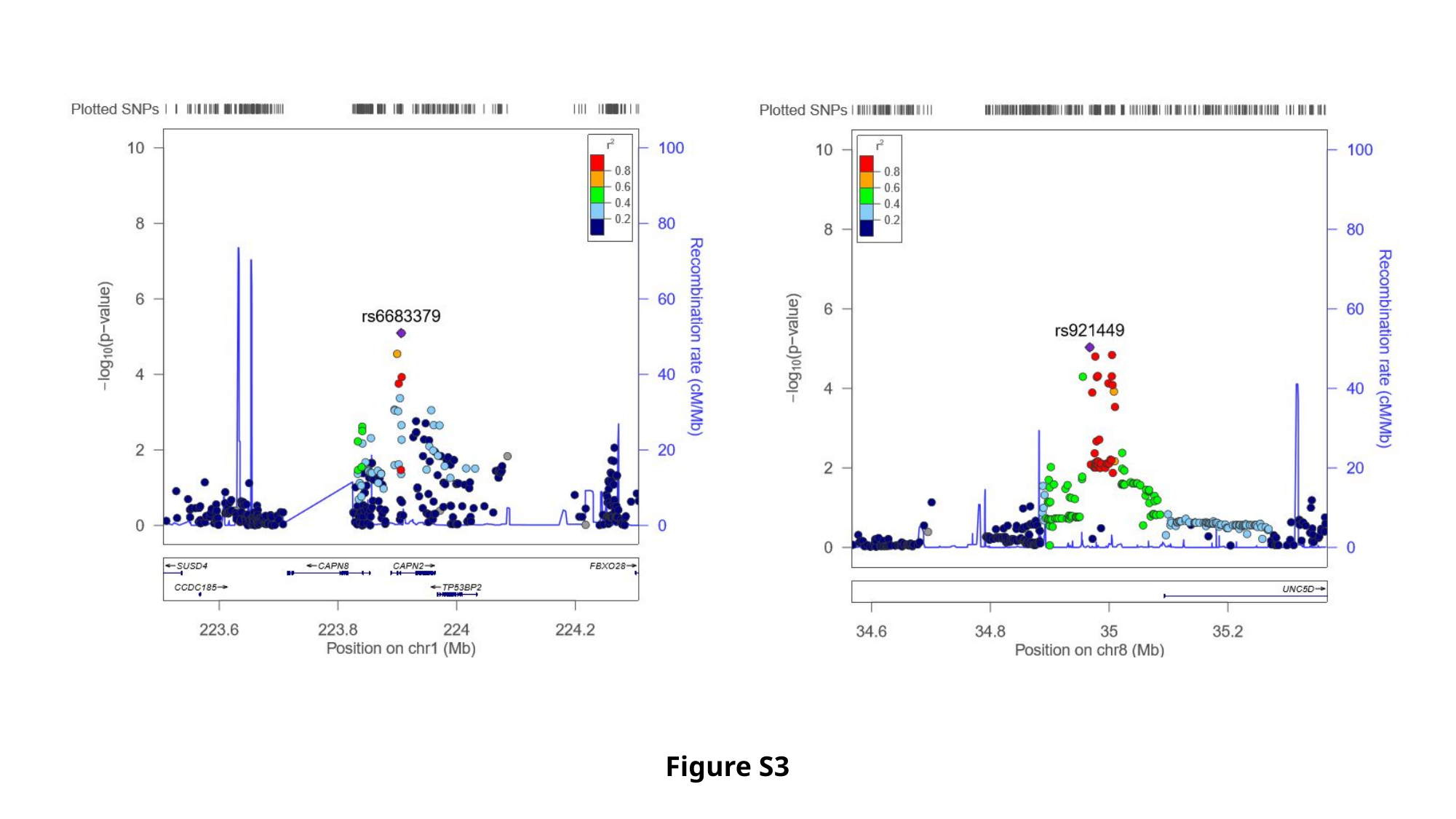

Figure S3

## Slide 10
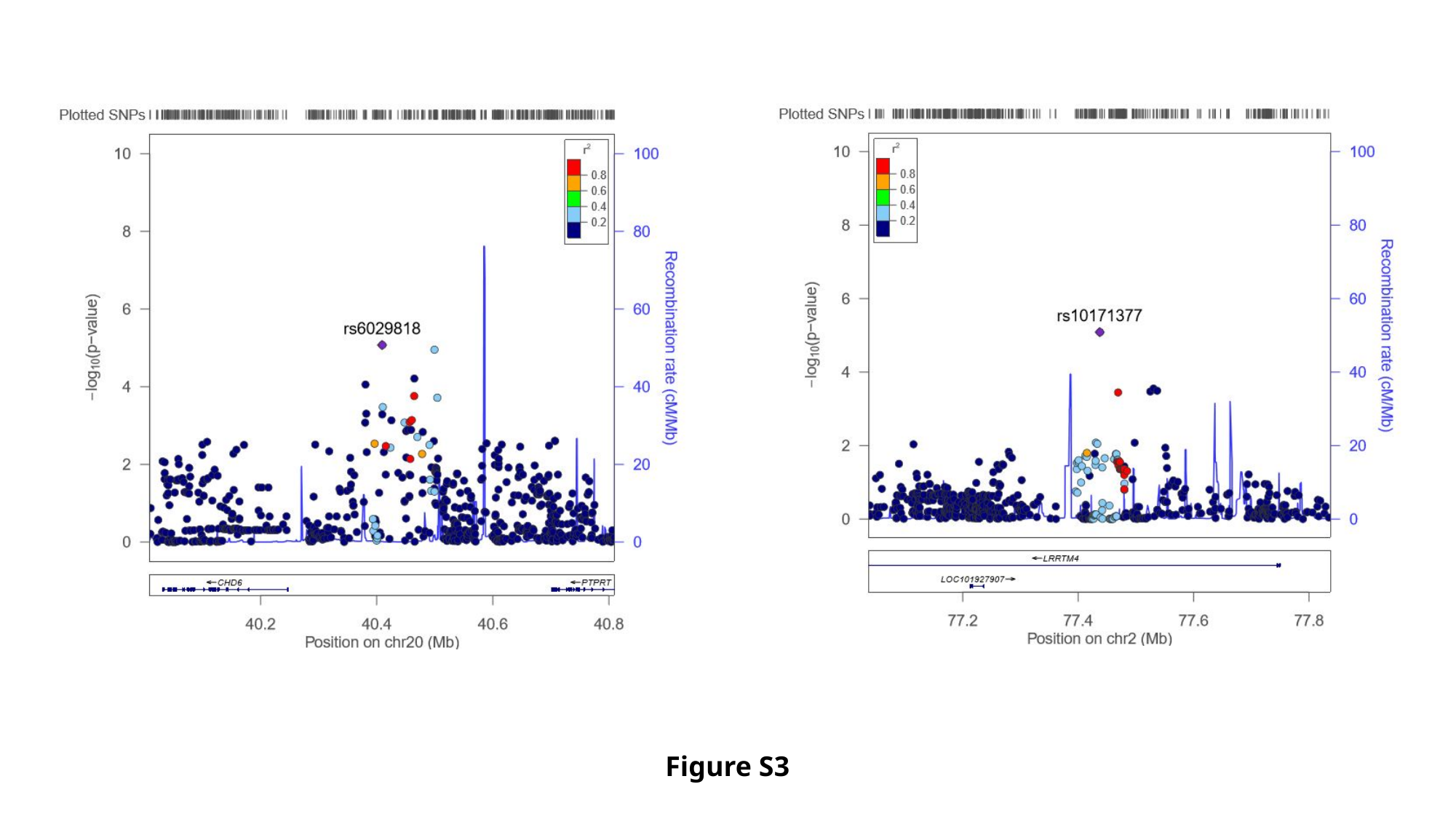

Figure S3

## Slide 11
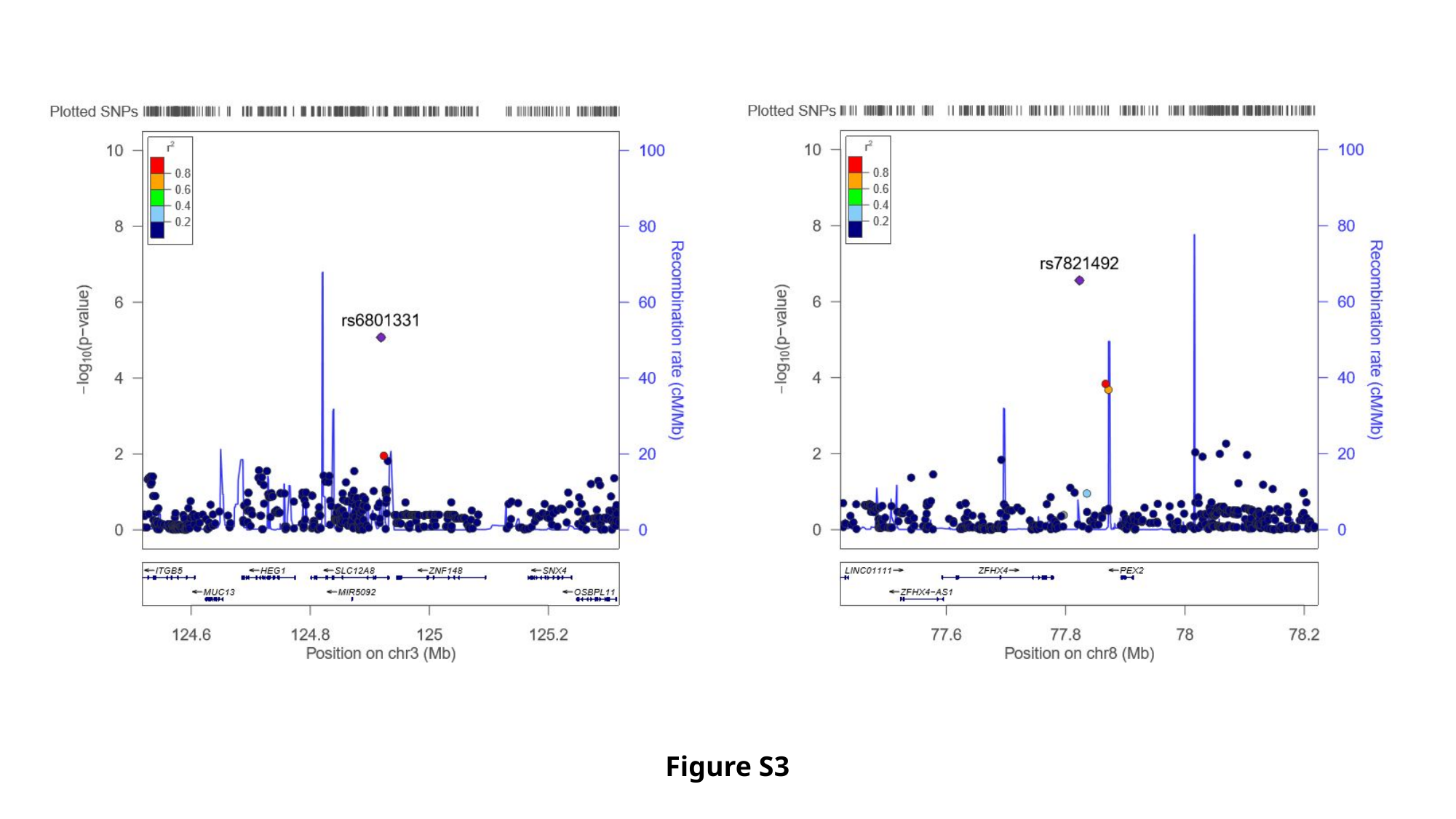

Figure S3

## Slide 12
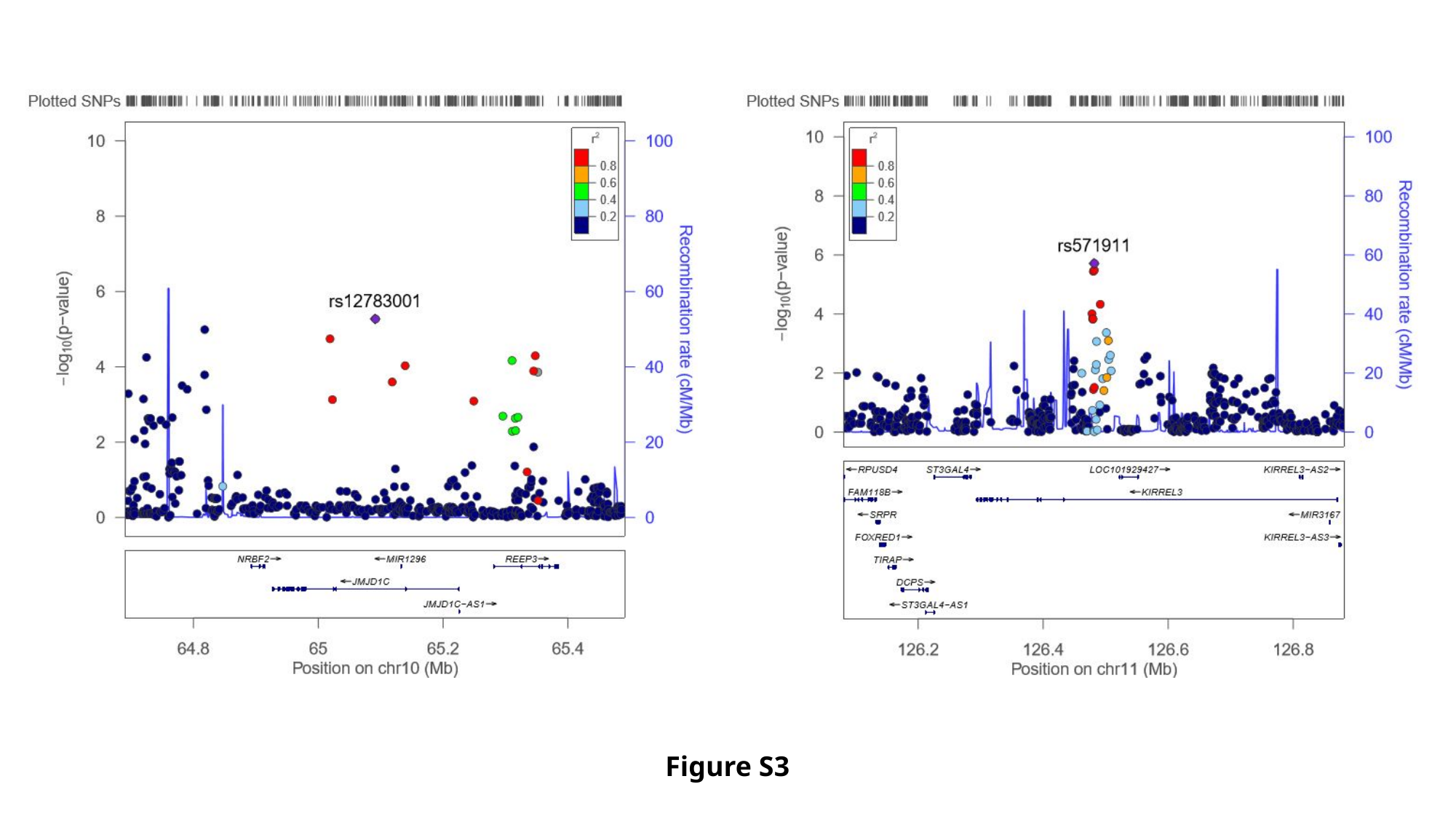

Figure S3

## Slide 13
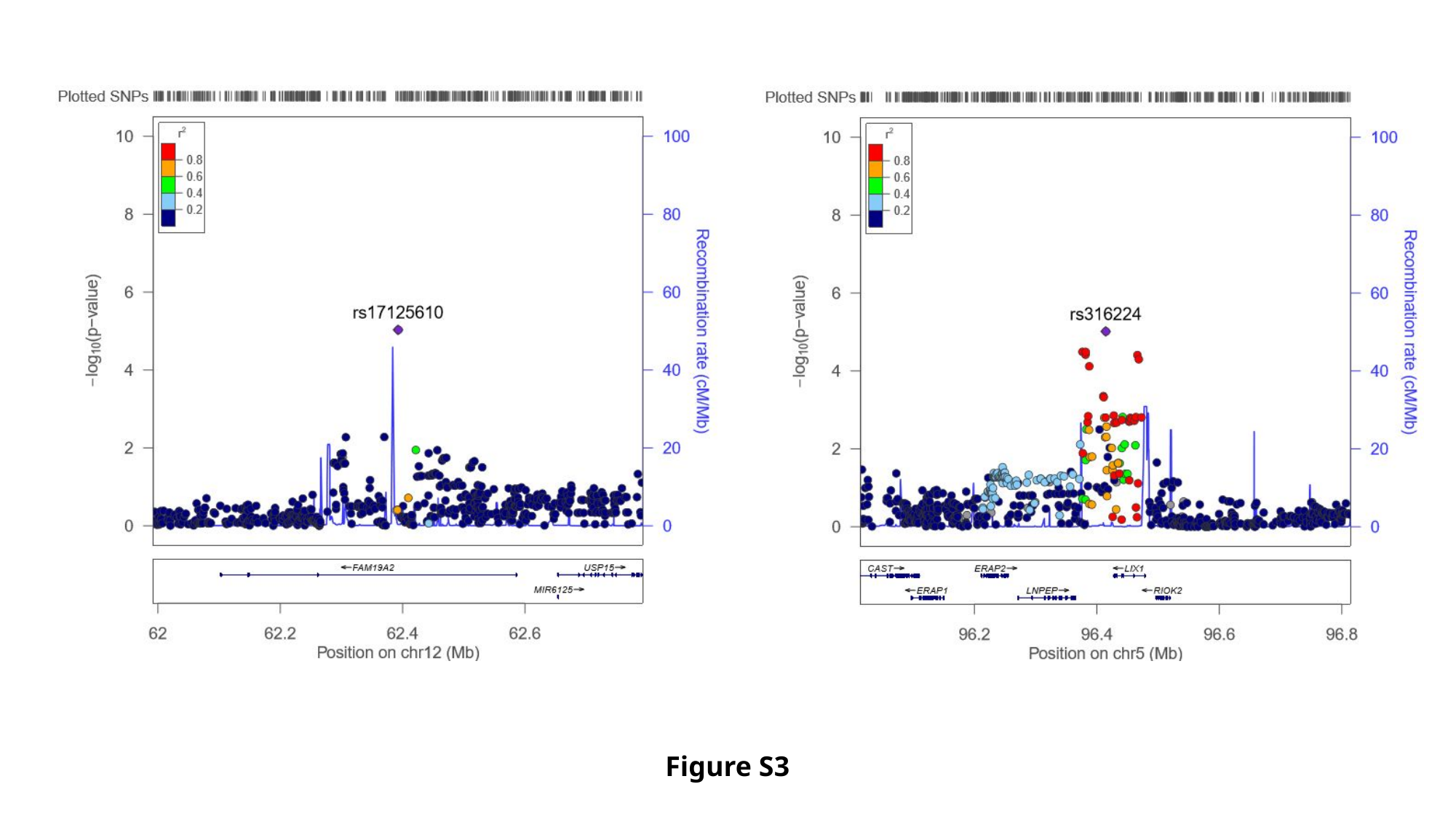

Figure S3

## Slide 14
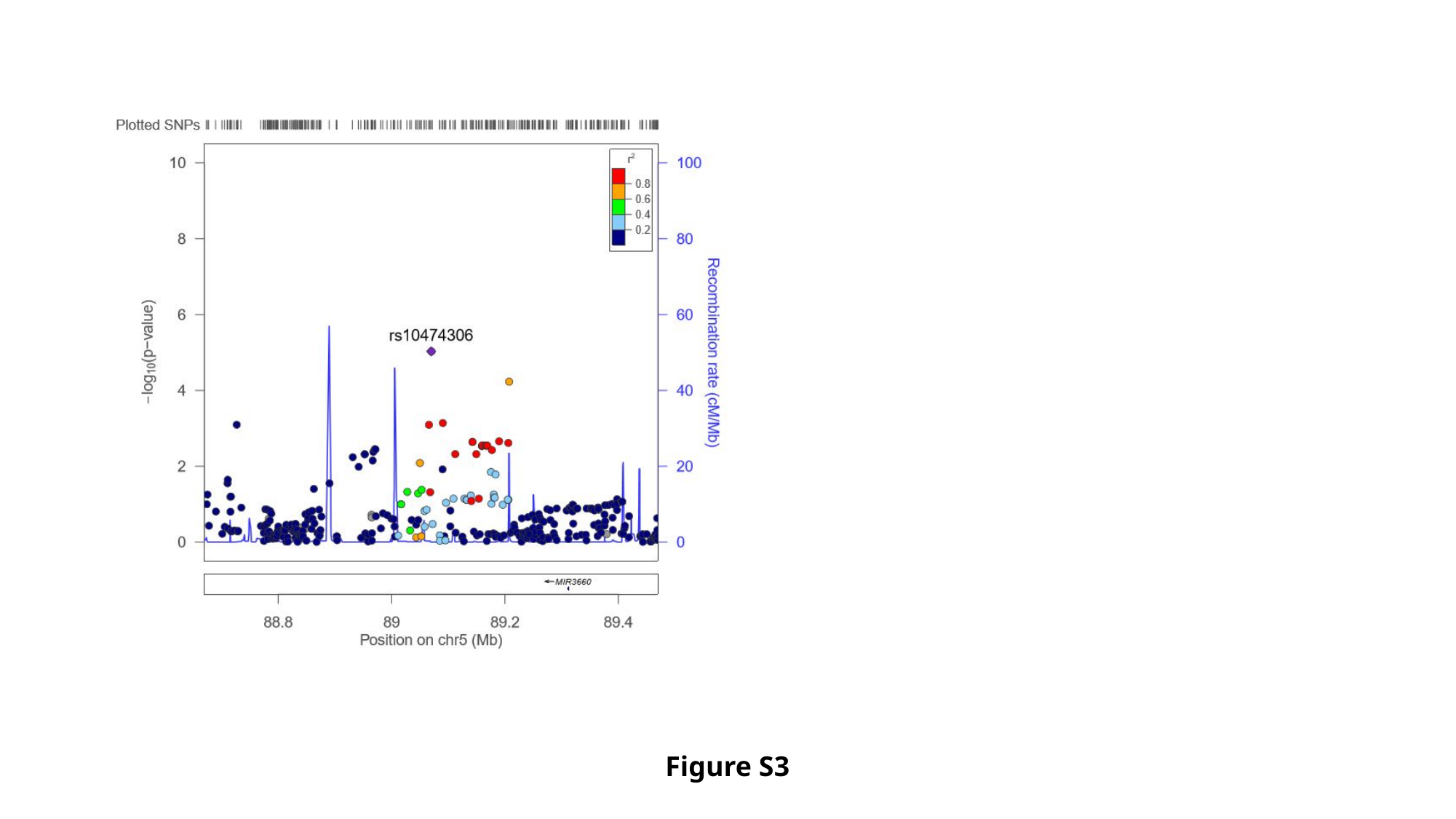

Figure S3

## Slide 15
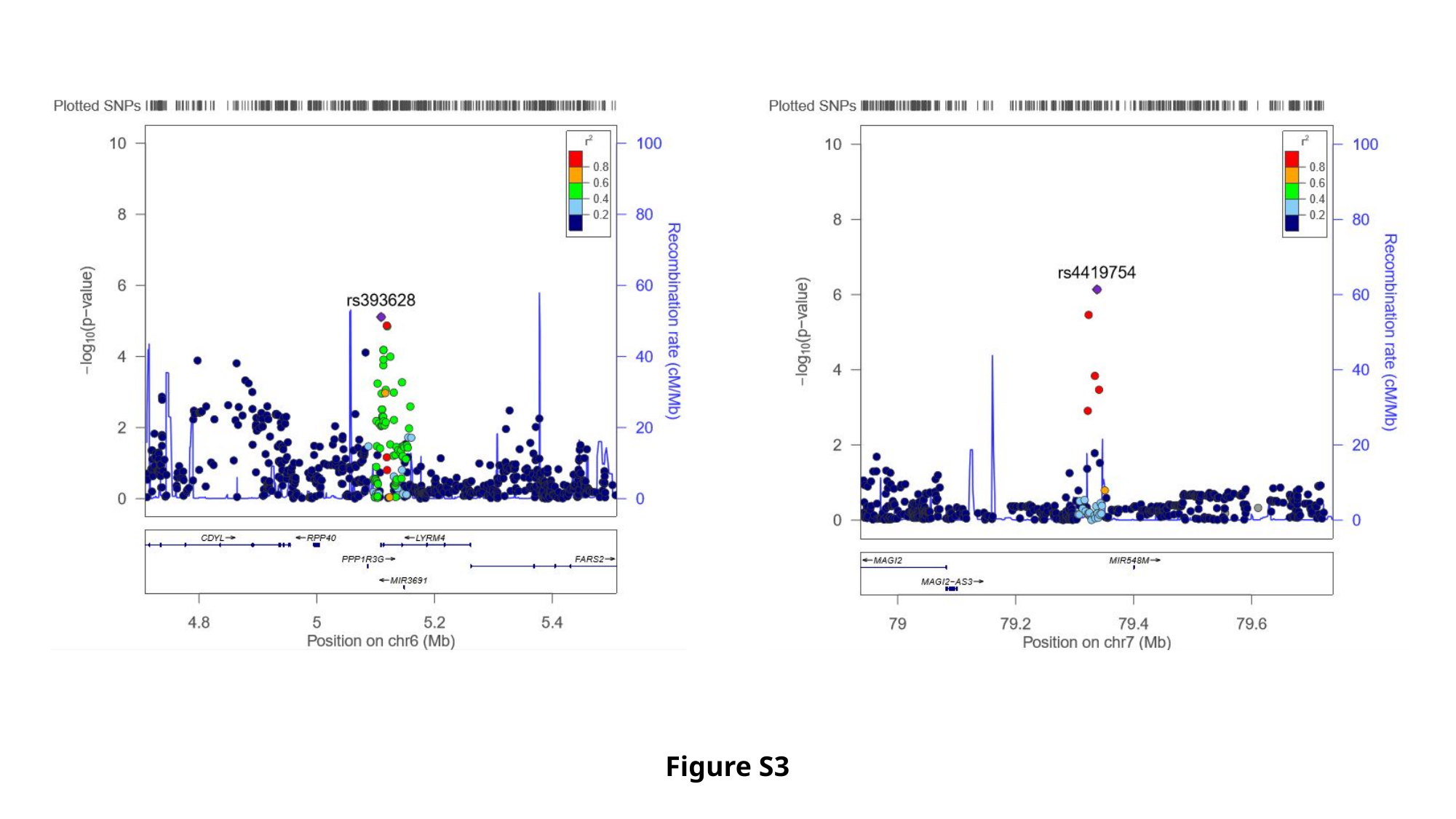

Figure S3

## Slide 16
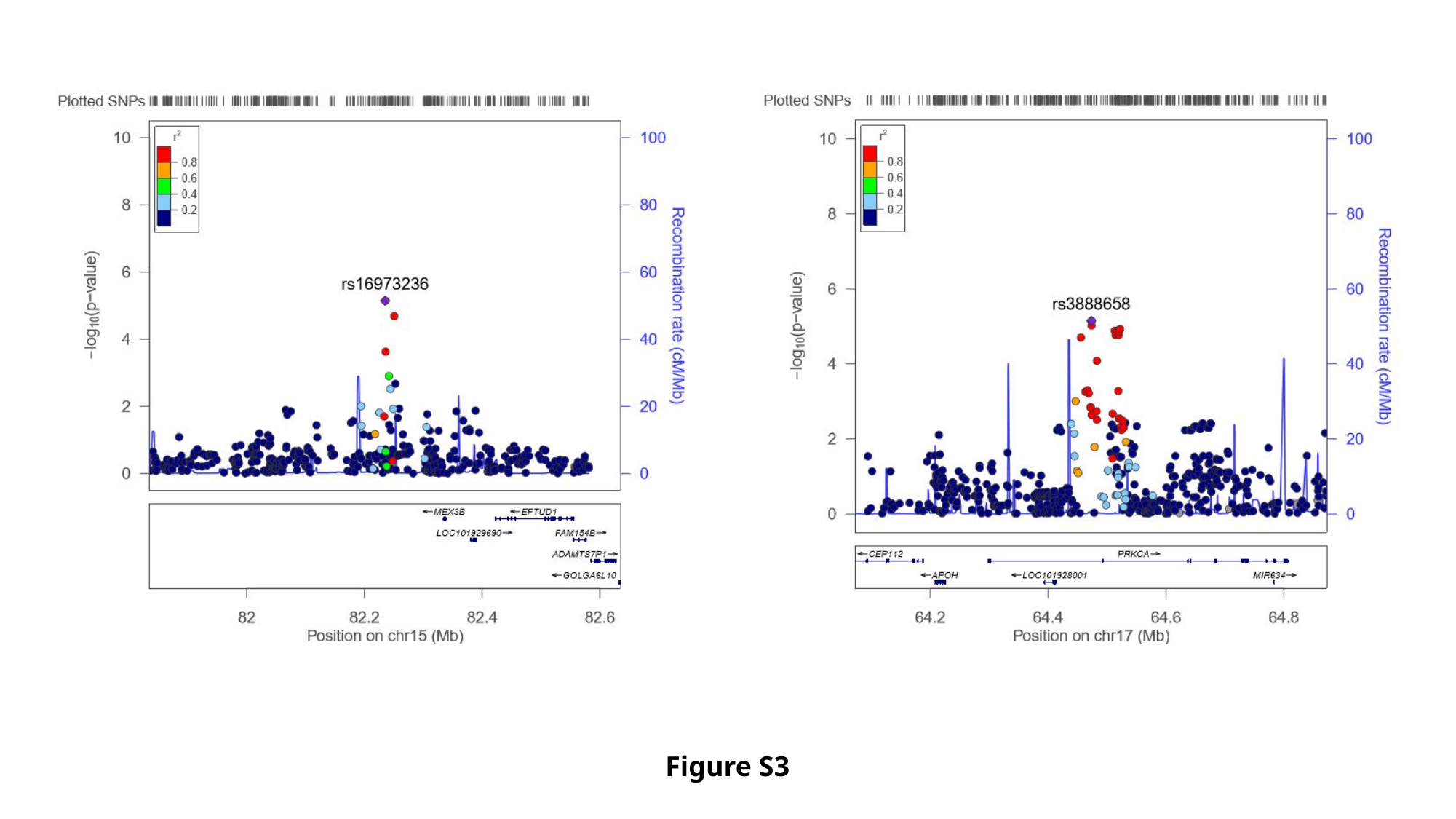

Figure S3

## Slide 17
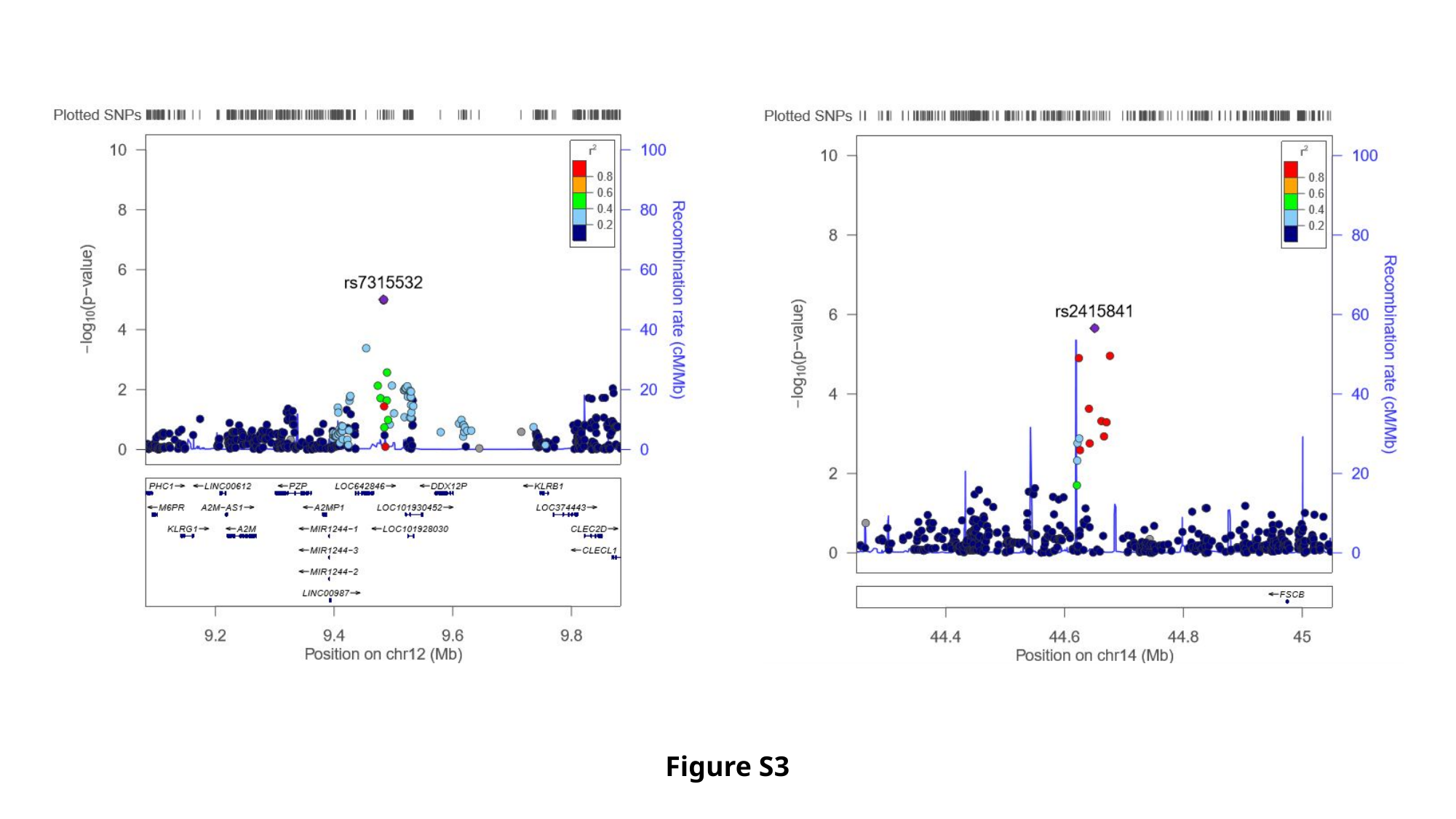

Figure S3

## Slide 18
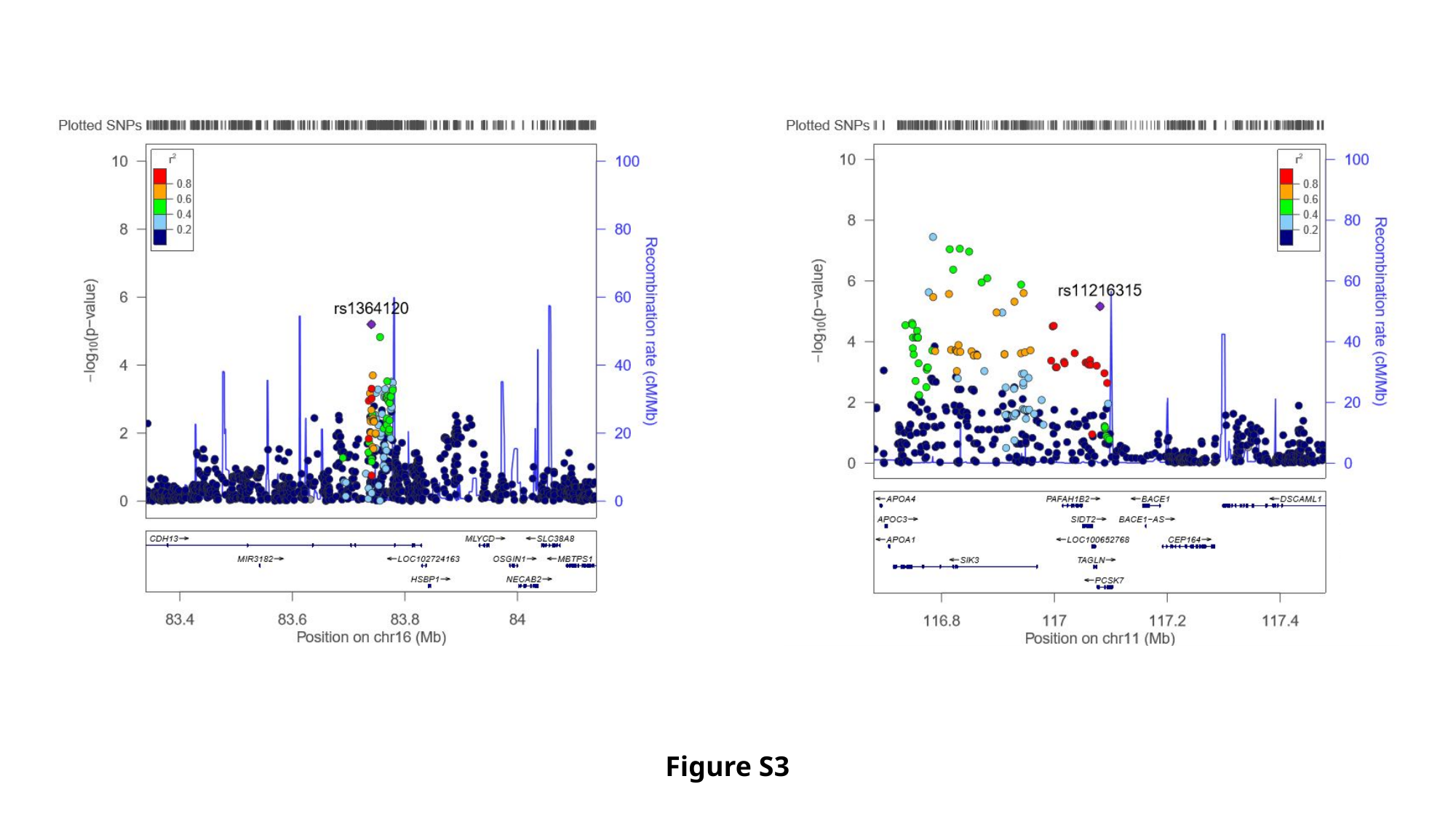

Figure S3

## Slide 19
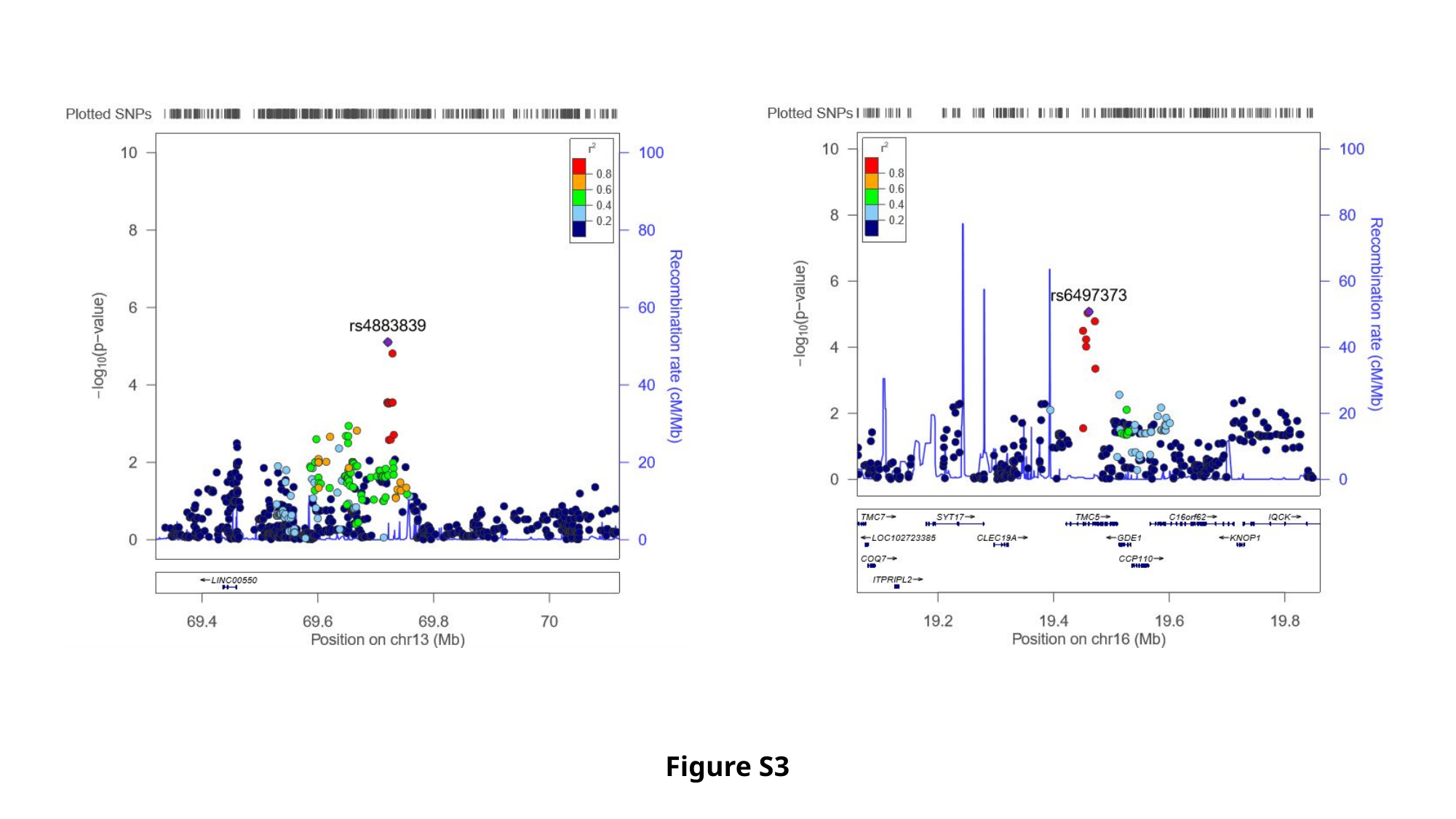

Figure S3

## Slide 20
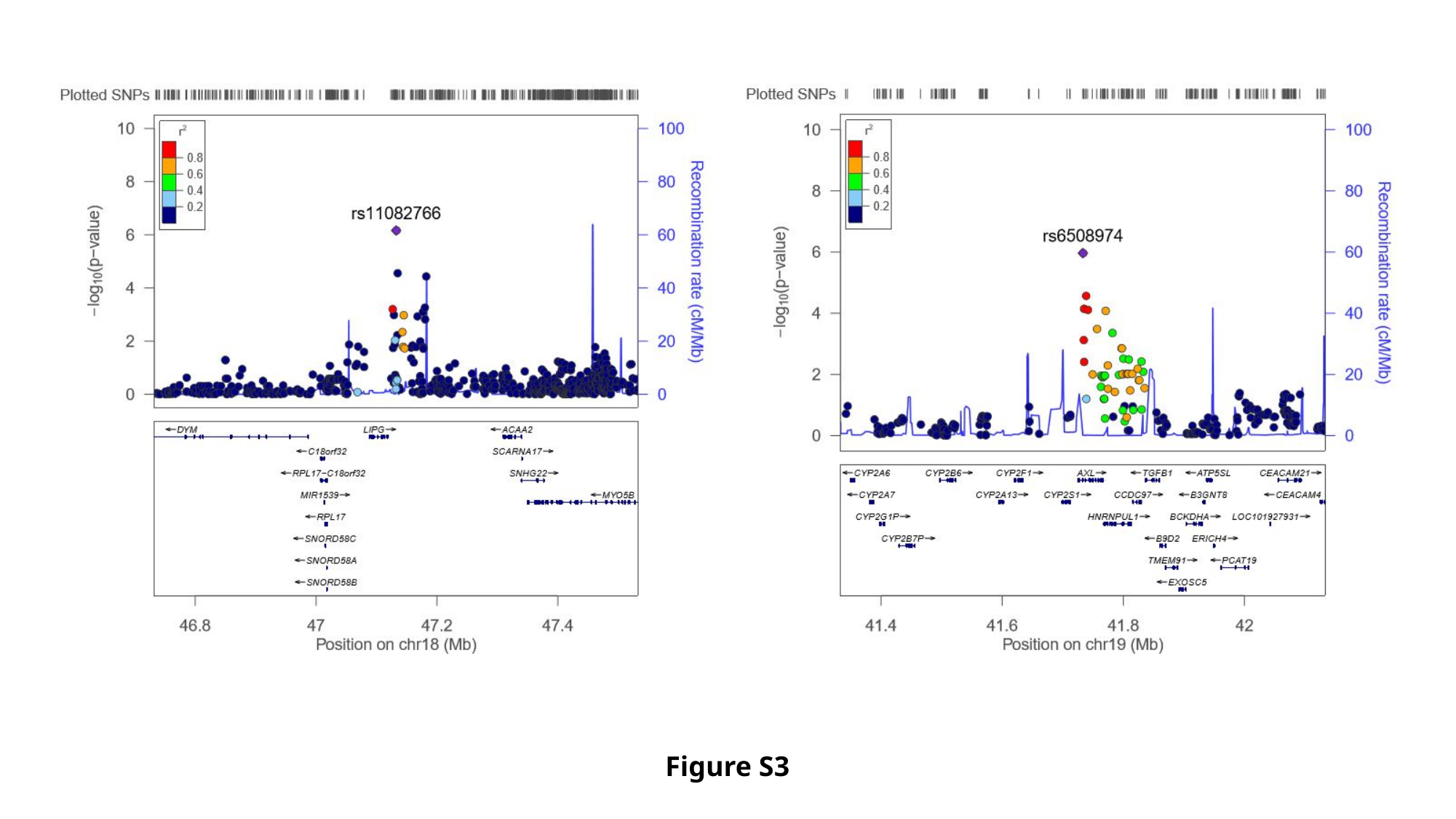

Figure S3

## Slide 21
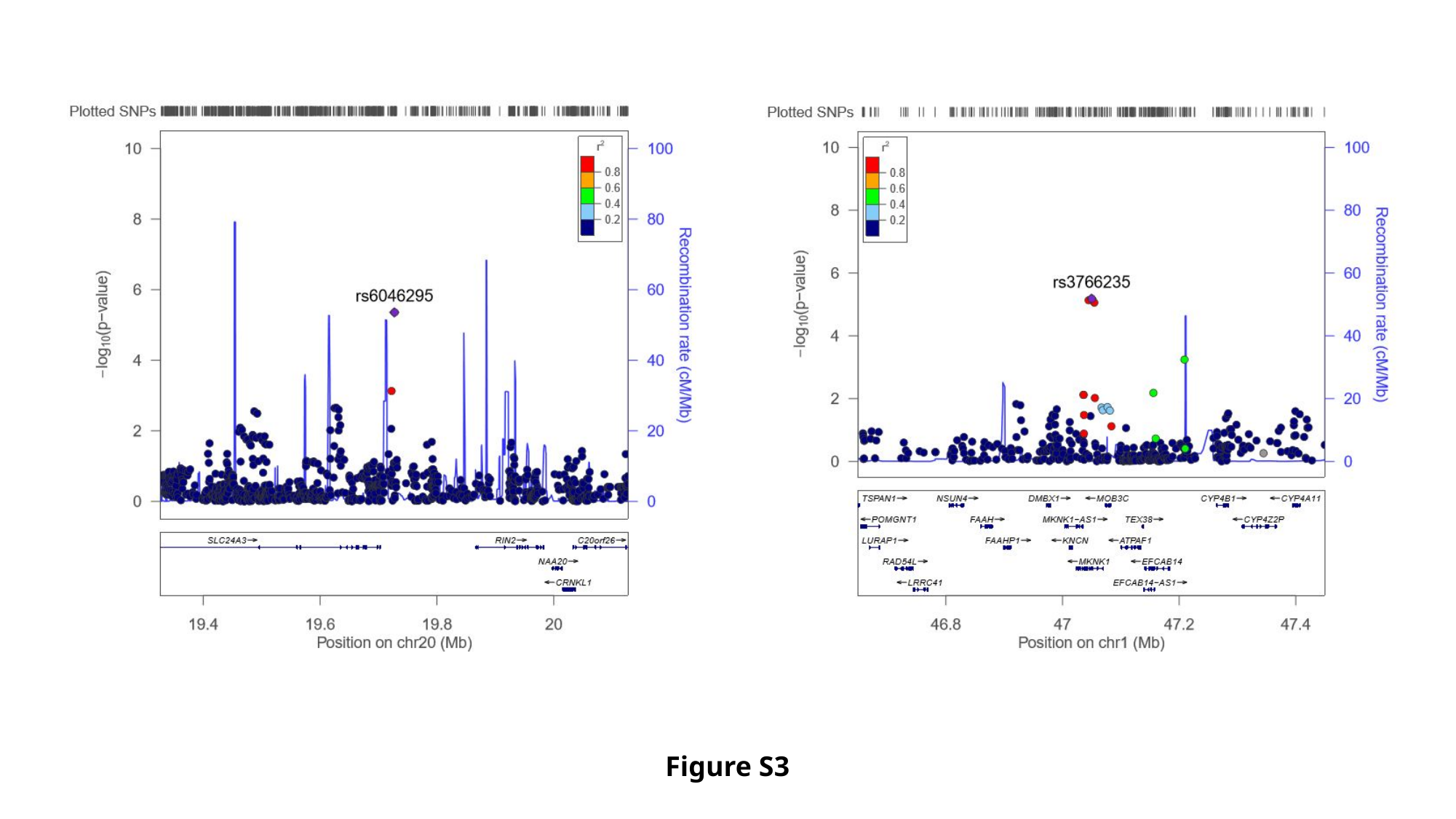

Figure S3

## Slide 22
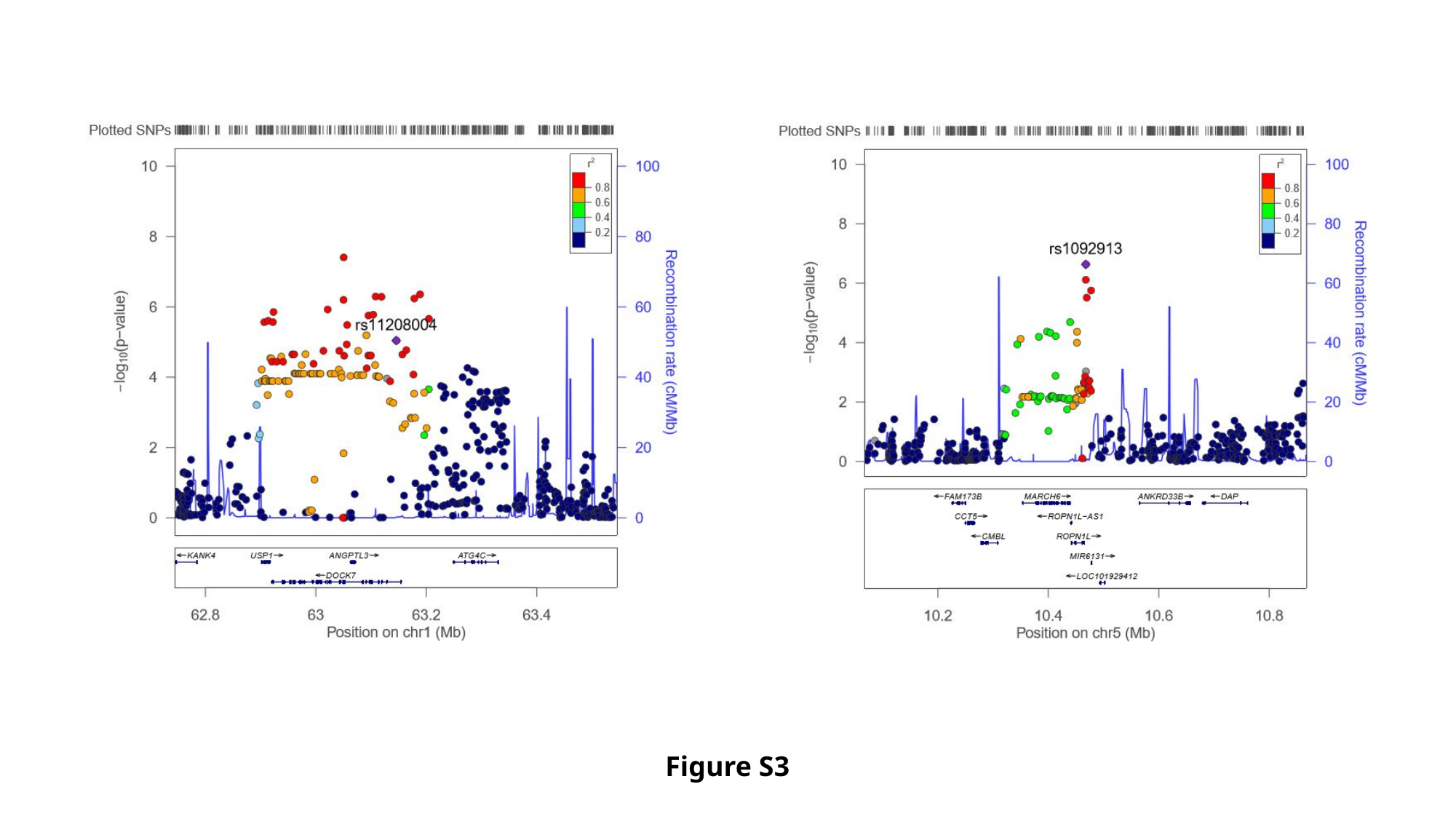

Figure S3

## Slide 23
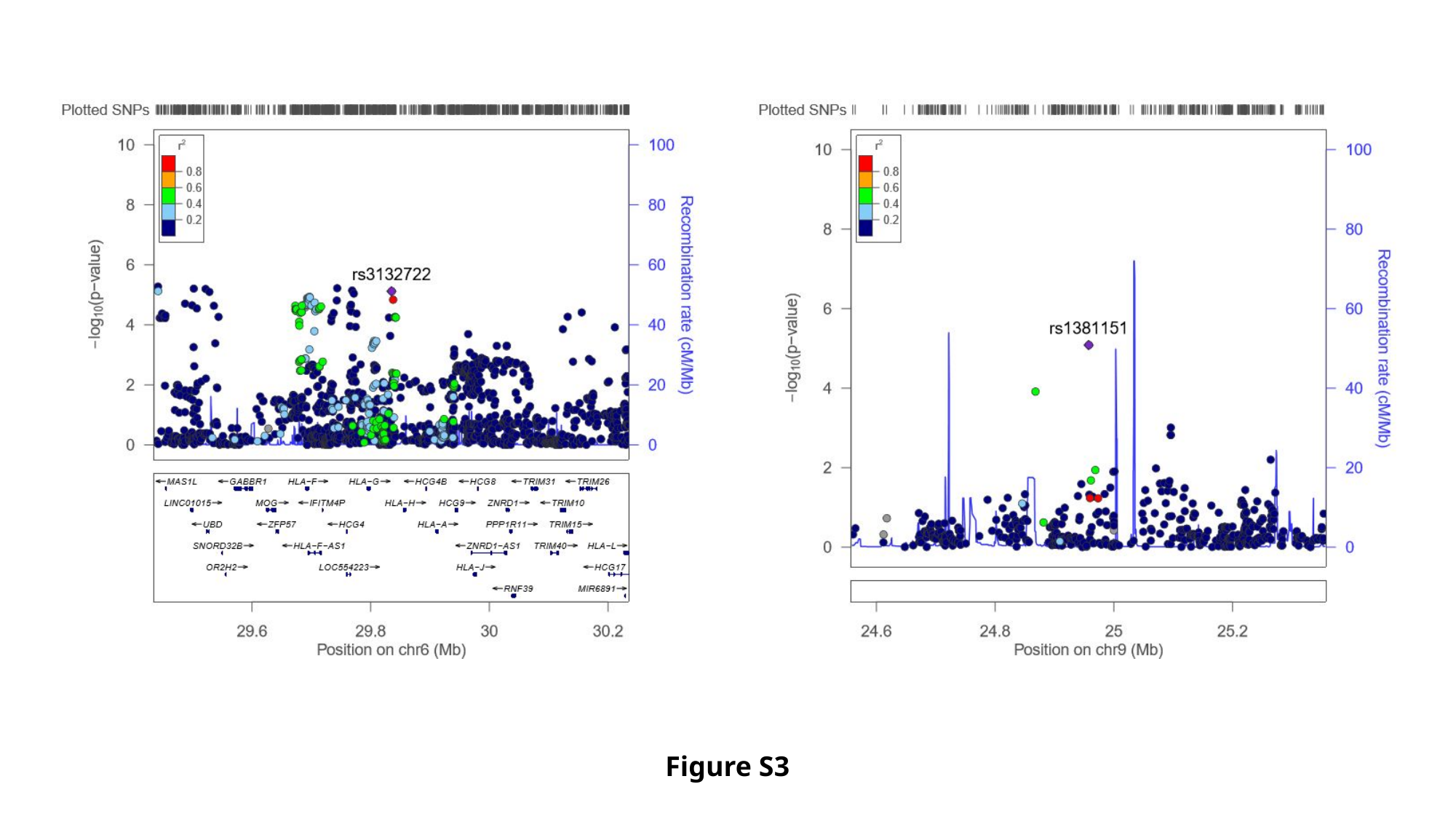

Figure S3

## Slide 24
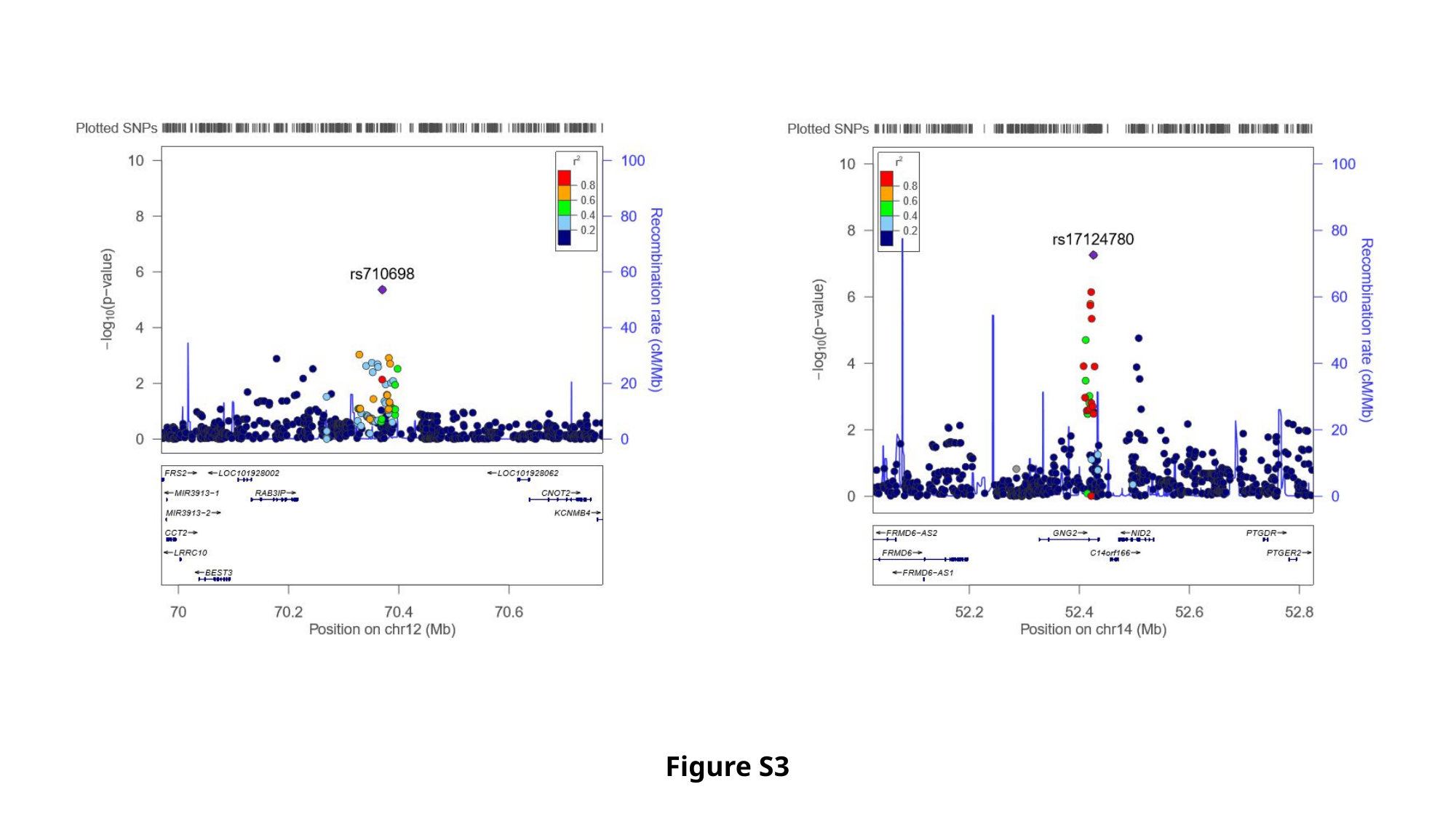

Figure S3

## Slide 25
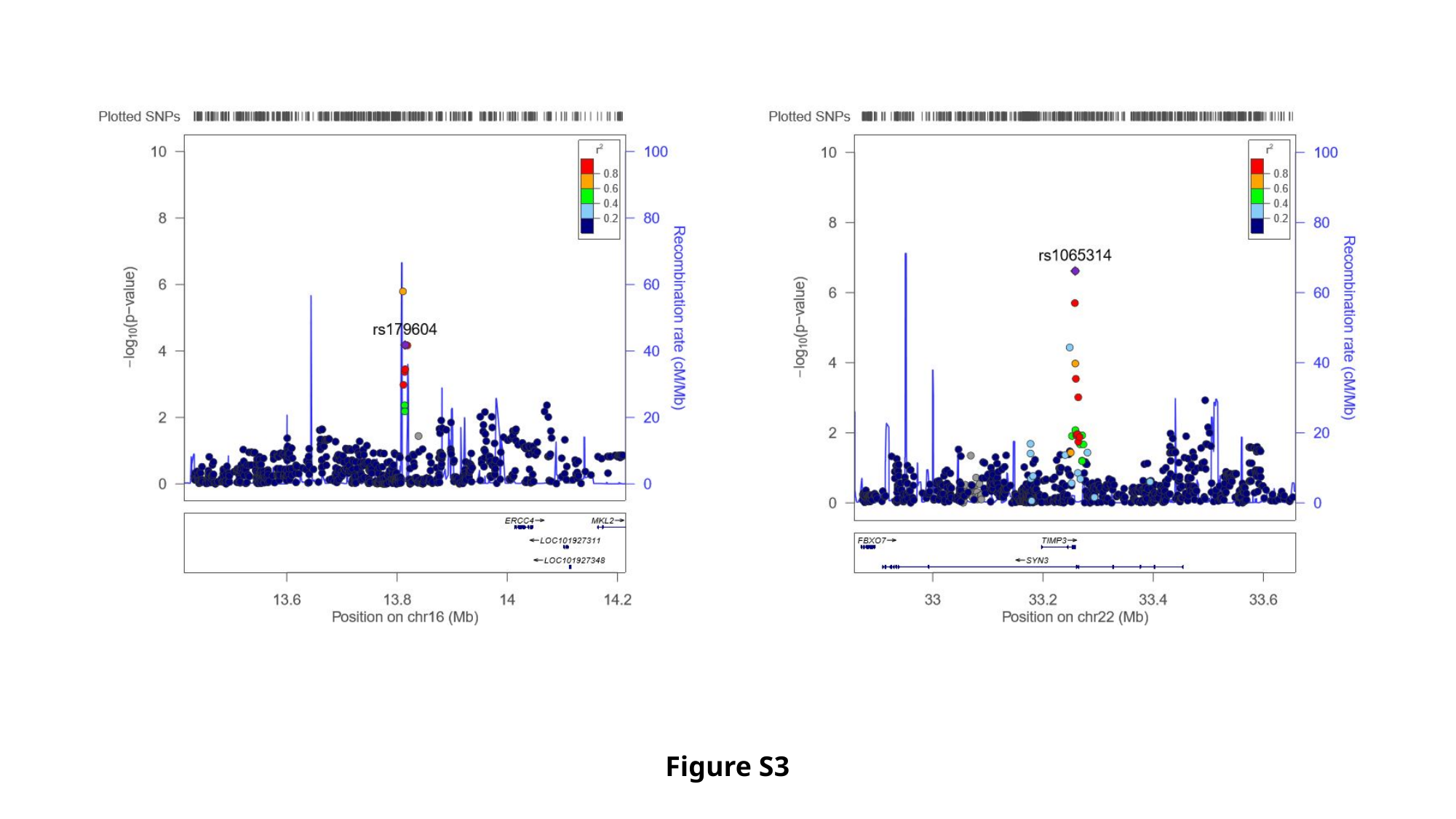

Figure S3

## Slide 26
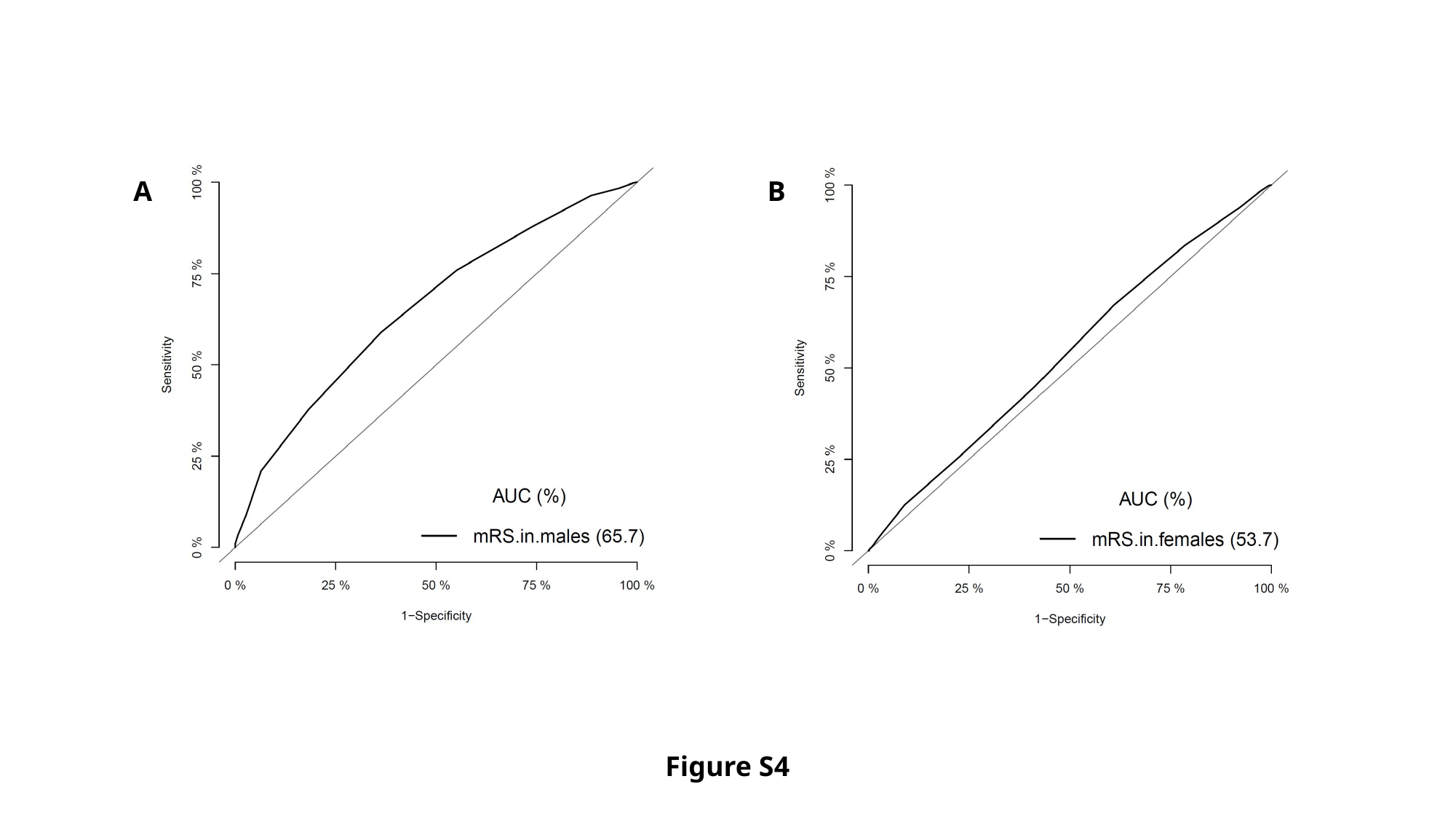

A
B
Figure S4
